# Enhancing *In Silico* Protein-Based Vaccine Discovery for Eukaryotic Pathogens Using Predicted Peptide-MHC Binding and Peptide Conservation Scores

**DOI:** 10.1371/journal.pone.0115745

**Published:** 2014-12-29

**Authors:** Stephen J. Goodswen, Paul J. Kennedy, John T. Ellis

**Affiliations:** 1 School of Medical and Molecular Sciences, University of Technology Sydney (UTS), Ultimo, NSW, Australia; 2 School of Software, Faculty of Engineering and Information Technology and the Centre for Quantum Computation and Intelligent Systems at the University of Technology Sydney (UTS), Ultimo, NSW, Australia; University of Ulm, Germany

## Abstract

Given thousands of proteins constituting a eukaryotic pathogen, the principal objective for a high-throughput *in silico* vaccine discovery pipeline is to select those proteins worthy of laboratory validation. Accurate prediction of T-cell epitopes on protein antigens is one crucial piece of evidence that would aid in this selection. Prediction of peptides recognised by T-cell receptors have to date proved to be of insufficient accuracy. The *in silico* approach is consequently reliant on an indirect method, which involves the prediction of peptides binding to major histocompatibility complex (MHC) molecules. There is no guarantee nevertheless that predicted peptide-MHC complexes will be presented by antigen-presenting cells and/or recognised by cognate T-cell receptors. The aim of this study was to determine if predicted peptide-MHC binding scores could provide contributing evidence to establish a protein’s potential as a vaccine. Using T-Cell MHC class I binding prediction tools provided by the Immune Epitope Database and Analysis Resource, peptide binding affinity to 76 common MHC I alleles were predicted for 160 *Toxoplasma gondii* proteins: 75 taken from published studies represented proteins known or expected to induce T-cell immune responses and 85 considered less likely vaccine candidates. The results show there is no universal set of rules that can be applied directly to binding scores to distinguish a vaccine from a non-vaccine candidate. We present, however, two proposed strategies exploiting binding scores that provide supporting evidence that a protein is likely to induce a T-cell immune response–one using random forest (a machine learning algorithm) with a 72% sensitivity and 82.4% specificity and the other, using amino acid conservation scores with a 74.6% sensitivity and 70.5% specificity when applied to the 160 benchmark proteins. More importantly, the binding score strategies are valuable evidence contributors to the overall *in silico* vaccine discovery pool of evidence.

## Introduction

An *in silico* protein-based vaccine discovery pipeline for eukaryotic pathogens, inspired by reverse vaccinology [1]–[6], encapsulates a collection of various bioinformatics prediction tools [7]. The aim of these tools is to gather computational evidence, derived mainly from protein sequences, to select the most promising vaccine candidates worthy of laboratory validation [8]. One piece of evidence, considered crucial in the candidacy decision making, is the presence of epitopes on protein antigens.

Many tools have been and are still being developed to computationally predict epitopes (see S1 Supporting Information). T-cell epitopes, which are typically short linear peptides, have proved to be easier to predict than B-cell epitopes [9]–[11]. Currently, there are two computational approaches to T-cell epitope prediction based on direct and indirect methods. A direct method predicts peptides recognised by T-cell receptors, whereas an indirect method predicts peptides binding to MHC molecules. Direct methods, as to date, have proved to be of insufficient accuracy [9] and this may be why the majority of T-cell epitope predictors currently found online are based on indirect methods. This paper focuses on the indirect method and the MHC class I molecule.

Most vaccines licensed so far are serum antibody-based that essentially provide protection from infection. Current opinion suggests that T-cell epitope ‘only’ vaccines are not a solution to prevent infection, but are important in controlling an established infection by the recognition and clearance of infected cells [10]. For many infectious diseases (and cancers) it remains an open question if cell-mediated immunity (CMI) is required for successful prevention or eradication, either in addition to or instead of antibodies [11].

**Figure 1:**
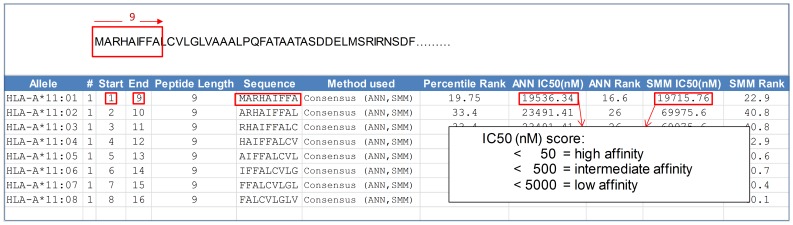
Example of online output from IEDB peptide-MHC class I binding predictor. The binding predictor conceptually slides a window of a user-defined length (either eight to eleven amino acid residues) one residue at a time from the start of the protein sequence. An affinity score is predicted for the ability of each fixed-length subsequence (as defined by each position of the sliding window) to bind to a user-specified MHC I allele. Fig. 1 shows the output when a sequence (e.g. MARHAIFFALCVLGL…) is input into the program to predict if it contains peptides of length 9 that bind to the MHC allele, HLA-A*11∶01. The IC_50_ (nM) affinity scores for subsequence ‘MARHAIFFA’ at position 1 to 9 are highlighted.

The foremost resource for T-Cell MHC class I binding prediction tools is provided by the Immune Epitope Database and Analysis Resource (IEDB) [12]. The MHC class I binding predictor (referred henceforth as the peptide-MHC binding predictor) takes as input an amino acid sequence (or a set of sequences) and predicts the binding affinity of each fixed-length subsequence to a specific MHC molecule. Fig. 1 shows an example of the online output. S1 Supporting Information describes the prediction process in detail including the methods used for computation.

The desired aim, from an *in silico* vaccine discovery perspective, is to use binding affinity scores as contributing evidence to a pool of other computationally derived evidence and, at this stage, not to specifically identify epitopes for vaccine development. The pool of evidence, in this instance, is ultimately used to support or oppose a protein as a candidate for a CMI-driven vaccine. Two important issues need to be emphasised here. First, there is no guarantee that a protein *predicted* to contain peptides that bind to a particular MHC allele will be presented by antigen-presenting cells and/or recognised by cognate T-cell receptors. Consequently, binding scores are only one piece of evidence and need to be used in conjunction with other gathered evidence as part of an overall vaccine candidate discovery strategy. Second, there are potentially thousands of proteins, mostly uncharacterised, constituting a pathogenic microorganism. An *in silico* discovery strategy demands an automated high-throughput process to extract evidence as it is impractical for a researcher to perform a case-by-case examination of each protein.

There are potentially thousands of pathogenic organisms for which vaccines are needed to improve human and animal health. Whilst advances in reverse vaccinology have begun to provide vaccines for prokaryotic pathogens [13]–[17], similar advances for eukaryotic pathogens such as parasites cannot be claimed. *Toxoplasma gondii*, a protozoan parasite and important model system for the phylum Apicomplexa [18]–[20], was chosen in this study to illustrate the presented strategies. The first main reason for choosing *T. gondii* is that the indirect prediction method is dependent on experimentally validated peptide-MHC binding data from the host of the target pathogen. Humans are the intermediate host for *T. gondii* in which it is responsible for birth defects and foetal loss [21]. Data for human MHC alleles, referred to as human leukocyte antigen (HLA), is by far the most abundant. The peptide-MHC binding predictor makes available 2947 MHC I alleles but distinguishes 76 of these alleles as commonly occurring in at least 1% of the human population (18 HLA-A, 32 HLA-B, 20 HLA-C, 1 HLA-E, 5 HLA-G). The second reason is that a large body of literature suggest that because *T.gondii* is an intracellular parasite, the most important correlate of protection is the induction of a CMI response (this is in addition to a humoral response) [22]–[26].

Predicted binding scores alone provide no direct indication of a protein’s potential to induce a T-cell response. The aim of this study is therefore to determine how these scores can best be utilised to provide such an indicator. We present two high-throughput classification strategies. Ideally, two types of protein examples are required to credibly demonstrate and test the strategies: proteins that are known to generate a T-cell response in the host (positives) and those known not to generate a response (negatives). The challenge for obtaining reliable positives is that there is no known example of an effective T-cell based vaccine, and even no clear consensus as to what type of protein constitutes an ideal vaccine candidate for a T-cell mediated response. Furthermore, no distinguishing signal within a protein sequence has been detected that indicates a protein not only contains T-cell epitopes but also induces an immune response. The challenge for negative examples is that a protein cannot be definitively negative unless it has been explicitly tested for non-inducement against all common MHC alleles. To face these challenges, the test and training proteins selected for the study are the optimum within the constraints of available data and current knowledge. For example, in the absence of what constitutes an ideal vaccine candidate, the selected proteins are only likely vaccine candidates – ‘likely’ in this context is based on *a priori* held hypotheses that a protein that is either external to or located on, or in, the membrane of a pathogen is more likely to be accessible to surveillance by the immune system than a protein within the interior of a pathogen [27]. Also, the majority of proteins in published studies observed to induce T-cell responses are membrane-associated or secreted [22], [28]–[34]. A few examples of proteins with annotated interior subcellular locations, nevertheless, have been observed to induce an immune response [33], which confounds the search for a typical T-cell inducing protein. To summarise, proteins naturally exposed to the immune system are considered in this study as potential vaccine candidates or positives, and unexposed proteins as non-vaccine candidates or negatives.

The first proposed strategy uses random forest, a supervised machine learning (ML) algorithm. The algorithm essentially detects patterns within a series of binding scores that are computed for the entire length of each protein. Quality examples of binding scores from positive and negative proteins are the key to the strategy i.e. they are required to train the ML algorithm. Once trained, the algorithm can distinguish likely vaccine or non-vaccine candidates given a new series of binding scores from an anonymous protein.

The second proposed strategy uses amino acid conservation scores of predicted binding peptides and is based on the principle that eukaryotic pathogens have evolved with the vertebrate immune system [35]. More specifically, amino acids of *T. gondii* proteins can change over time under the evolutionary selection pressure of the human immune system. For example, human HLA alleles evolve to optimise fitness and *T. gondii* antigens adapt to evade HLA capture. Amino acid residues that are vital to a protein’s function tend to be the most highly conserved in homologous proteins as they change less rapidly during evolution. This means that *T. gondii* antigens are in an evolutionary balancing act to evade the immune system by varying their antigens but still retaining functionality. It is suggested that immune evasion is achieved by changes at multiple sites rather than clustering polymorphisms at one site [36]. In other words, amino acid sites in a protein antigen are expected to be under different selection pressures and their conservation will vary accordingly. There are examples of apicomplexan proteins where amino acids exposed to the immune system are more polymorphic than those unexposed [37]–[39]. We speculate that binding peptides will be located more often in less conserved sites. However, from an epitope-based vaccine perspective, conserved epitopes are more desirable to provide broad protection across multiple strains [40]. A multiple sequence alignment (MSA) can be used to compare amino acid similarity between a protein and its homologues [41]. The degree of residue conservation is inferred from the similarity within an aligned column. Various methods have been developed to quantitatively score the conservation and are compared in a review [42]. Our strategy compares conservation scores of predicted binding peptides to distinguish between potential vaccine or non-vaccine candidates.

We conclude that there is no single program that can predict proteins that will elicit T-cell immune responses. The current best approach is to obtain a consensus from several programs to select those proteins ‘most likely’ to induce the required response. The results obtained from the ML and conservation strategies are not perfect owing to constraints posed by the programs and available data. Nevertheless, the strategies still provide valuable evidence towards a consensus. Currently, there is no known alternative solution for using binding scores in a high-throughput approach to identify vaccine candidates.

## Results and Discussion

The first important question to answer was how accurate are the IEDB peptide-MHC class I binding predictions. Our results from an affinity-strength accuracy test revealed a sensitivity and specificity of 63.1% and 78.9% respectively. This indicates that the predictor has a relatively low sensitivity in predicting precise affinity strength. It has a greater sensitivity, however, to high-affinity peptides (e.g. 80.1%) than intermediate (45.4%) or low affinity (35.5%) peptides. The accuracy to predict binding or non-binding peptides, irrespective of affinity strength, was 91.7%. Other studies also report that the predictions are good, particularly for well-studied class I MHC alleles [43], [44].

Binding studies show that HLAs are the most polymorphic human genes known [45] and each HLA allele recognizes a restricted set of peptides [46]. Our results from a binding test on the entire known proteome of *T. gondii* (including different strains) were therefore not totally unexpected given the known polymorphic nature of MHC: 19355 out of 19378 proteins tested contained at least one high-affinity binding peptide. No predictions were made for 23 proteins as their sequence contained characters such as ‘X’ or ‘B’, which are non-standard to the amino acid single letter code and invalid to the predictor. To support this finding, binding predictions were analysed from 124 proteins from *Plasmodium falciparum* (an apicomplexan that can cause malaria) and 760 proteins from *Caenorhabditis elegans* (a non-pathogenic nematode). The important conclusion inferred from these results is that every protein from a eukaryotic pathogen is *predicted* to contain at least one peptide that binds with a high-affinity to at least one of the known human MHC I alleles. Given this finding, it is impracticable to select a protein for vaccine candidacy on the basis it contains a high-affinity peptide.

The output from the binding predictor are potentially thousands of IC_50_ (nM) scores for each protein under consideration. Table 1 shows descriptive statistics for predicted high-affinity peptides from the species *T. gondii*, *P. falciparum*, and *C. elegans.* The number of peptides range for *T. gondii* from five to 3768 per protein and each protein can contain peptides that bind to as few as six and to as many as 137 different allele-peptide length combinations.

**Table 1:** Descriptive statistics for predicted high-affinity peptides against 76 common human MHCs.

Description	Benchmark Proteins^a^	*P. falciparum*	*C. elegans*	*T. gondii* proteome
Number of proteins tested	160	124	760	19378
IC_50_ scores per protein	H = 4.5, L = 0.2, A = 1.45	H = 3.1, L = 0.2, A = 1.2	H = 7.7, L = 0.12, A = 1.3	H = 49.3, L = 0.03, A = 2.6
Number of peptides on a protein	Max = 1583, Min = 21, A = 292	Max = 2071, Min = 54, A = 350	Max = 2528. Min = 4, A = 354	Max = 3768, Min = 5, A = 227
Number of allele-peptide length combinations used per protein out of 304 combinations	Max = 283, Min = 21, A = 78	Max = 148, Min = 66, A = 81	Max = 330, Min = 21, A = 86	Max = 137, Min = 6, A = 66
Frequency of prediction method used	SMM = 124, ANN = 35, NetMHCpan = 1	SMM = 95, ANN = 27	SMM = 582, ANN = 173, NetMHCpan = 5	SMM = 14802, ANN = 2407, NetMHCpan = 2058
Maximum number of **proteins** with peptides that bind to the same peptide-allele length combination	28 (HLA-C*03∶03 length 11)	20 (HLA-C*14∶02 length 8)	98 (HLA-C*03∶03 length 10)	3515 (HLA-A*68∶01 length 9)
Maximum number of **peptides** that bind to the same peptide-allele length combination	1526 (HLA-C*03∶03 length 10)	1556 (HLA-C*14∶02 length 8)	7542 (HLA-C*03∶03 length 10)	406529 (HLA-B*58∶01 length 10)

Abbreviations: *P. falciparum*  =  *Plasmodium falciparum*, *C. elegans*  =  *Caenorhabditis elegans*, *T. gondii*  =  *Toxoplasma gondii*, H  =  highest, L  =  lowest, A  =  average, Max  =  maximum, Min  =  minimum, SMM  =  stabilized matrix method, ANN  =  artificial neural network.

a Benchmark Proteins are proteins from published studies with known or expected T-cell responses (source species: *T. gondii*).

### Results for rule-based classification

**Figure 2:**
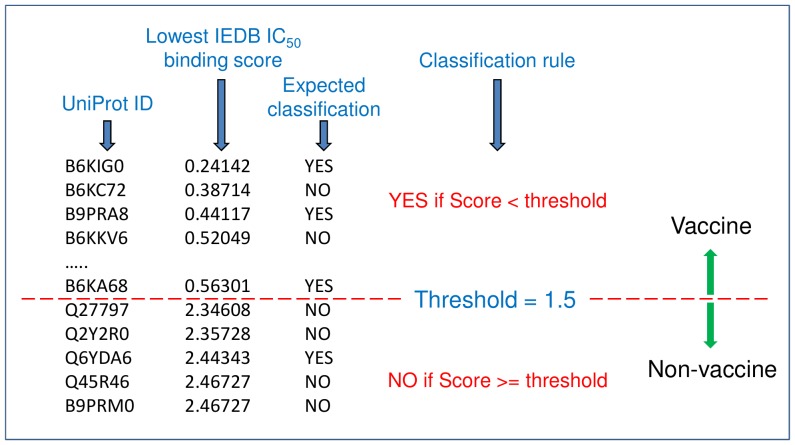
Example of rule-based approach applied to highest affinity peptide on each test protein. Proteins are listed in ascending order based on the lowest IC_50_ (nM) binding affinity score. A threshold value e.g. 1.5 is applied to the score to segregate the list into two classifications. Below the threshold is ‘YES’ for vaccine candidacy and above is ‘NO’. The rule-based classification is compared with the expected classification to determine performance accuracy. Threshold value is derived from a trial-and-error approach with the intention to classify the greatest number of true positives and negatives.

Rule-based tests were performed on a benchmark dataset to differentiate ‘YES’ from ‘NO’ vaccine candidates. That is, binding scores were ordered on different statistical properties of the score and then appropriate test thresholds applied to perform a binary classification. S2 Supporting Information lists the benchmark proteins along with a publication reference to the relevant study and provides a brief description of the vaccine significance for some of these proteins. Fig. 2 shows an example of the rule based-approach. The premise was to formulate decision making rules that could be applied to scores from anonymous proteins for the purpose of vaccine classification. The results are shown in Table 2. Accuracy around 50% is no better than guesswork in binary classifications with equally likely classes. Some of the rules showed promise but failed when applied to different datasets. These results suggest that both vaccine and non-vaccine candidates contain high-affinity binding peptides, peptides that bind to the same MHC allele, have similar numbers of binding peptides and promiscuous peptides per protein, and also have similar numbers of peptides that bind to promiscuous MHCs. The important conclusion here is that there is no universal set of rules that can be applied directly to binding scores to distinguish a vaccine from a non-vaccine candidate.

**Table 2:** Sensitivity and specificity for rule-based tests applied to high-affinity peptide-MHC binding scores for vaccine classification.

Rule #	Statistical property for rule-based test^a^	Threshold^b^	BenchmarkProteins^c^		*P.* *falciparum*		*C.* *elegans*	
			SN	SP	SN	SP	SN	SP
1	Lowest IC_50_ score per protein	1.5	42.7	45.8	69.8	32.0	60.9	38.3
2	Number of high-affinity peptides per protein	200	64.0	61.2	42.4	80.0	28.4	68.9
3	Number of different MHC alleles per protein binding to high-affinity peptides	74	56.0	63.5	43.8	79.2	25.5	73.5
4	Maximum number of high-affinity peptides per protein binding to a particular MHC allele-peptide length combination	10	66.7	58.2	47.9	69.4	37.3	65.3
5	Total binding score per protein	32289	61.3	61.2	58.9	72.0	36.8	59.1
6	Groups: one with proteins containing peptides binding to promiscuous MHCs; one with proteins containing peptides NOT binding to promiscuous MHCs	Not applicable	47.2	44.5	45.2	48.3	47.2	45.3

Abbreviations: *P. falciparum*  =  *Plasmodium falciparum*, *C. elegans*  =  *Caenorhabditis elegans*, *T. gondii*  =  *Toxoplasma gondii*, SN  =  sensitivity (%)  =  true positives/(true positives+false negatives), SP  =  specificity (%)  =  true negatives/(true negatives+false positives).

a Proteins ordered on statistical property and test thresholds applied to perform a binary classification.

b Threshold derived from a trial-and-error approach, using the mean as a seed threshold, on benchmark proteins to achieve the greatest number of true positives and negatives. Same universal rule (i.e. threshold) is applied to *P. falciparum* and *C. elegans* data.

c Benchmark Proteins are proteins from published studies with known or expected T-cell responses (source species: *T. gondii*).

### Results from supervised machine learning classification

**Figure 3:**
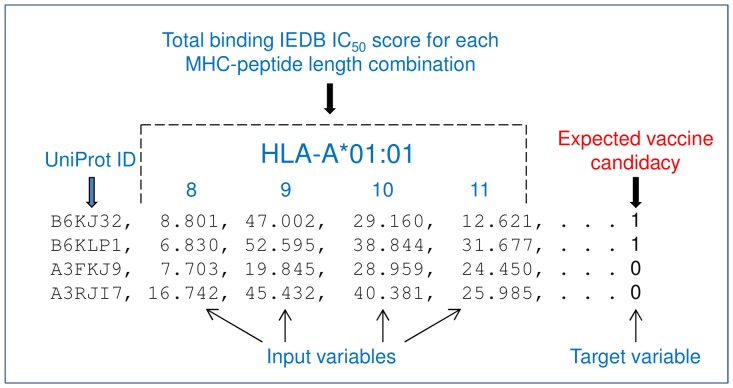
Example file format of training dataset used in machine learning. There is one protein per line that consists of the total binding affinity score for each peptide-MHC length combination e.g. 304 combinations for 76 common MHC I alleles (MHC I binds to peptides, typically eight to eleven amino acid residues in length. Therefore, 76 alleles * 4 peptide lengths  = 304 combinations). Binding affinity score  =  an IEDB IC_50_ (nM) score <5000. Each score is weighted by the length of the protein. The scores represent input variables or predictors. The last column is a 1 or 0 that indicates an expected ‘YES’ or ‘NO’ vaccine candidacy and represents the target variable. This expectation is based on the subcellular location annotation associated with the protein in UniProtKB (secreted or membrane-associated  = 1, internal location  = 0).

The best classification result achieved using random forest when trained on apicomplexan proteins was 72% sensitivity (SN) and 82.4% specificity (SP) with a 22.5% overall error rate. These results have the potential to improve when more training examples become available for proteins observed to induce, ideally protective, T-cell responses. Fig. 3 illustrates a training dataset. Table 3 shows results for all tests conducted with random forest. These results suggest that predictions improve when the source of training data is more closely related to the target pathogen. Additional tests were performed to determine if these promising results were achieved by chance. The value for the target variable (e.g. 1 or 0) in the training datasets was randomly set for each protein and the same tests were performed as before. The predictions, as shown in Table 3, are considerably less accurate, which supports the conjecture that there is a relationship between scores and target variable.

**Table 3:** Sensitivity and specificity for random forest tests applied to peptide-MHC binding scores for vaccine classification of Benchmark dataset.

Training dataset	Cross-validation^a^			Benchmark^b^		
	SN	SP	HE	SN	SP	OE
***Plasmodium falciparum***	**75.0**	**85.6**	**20.0**	**77.3**	**50.6**	**36.9**
*Plasmodium falciparum* (R)	38.9	61.7	49.7	58.2	49.7	54.2
***Caenorhabditis elegans***	**63.4**	**57.5**	**39.4**	**36.0**	**56.5**	**60.0**
*Caenorhabditis elegans* (R)	55.4	48.3	51.6	52.4	56.4	54.3
**Apicomplexans**	**86.7**	**80.7**	**16.1**	**72.0**	**82.4**	**22.5**
Apicomplexans (R)	54.0	38.2	42.9	74.0	39.0	42.5

Abbreviations: (R)  =  target variable e.g. 1 or 0 in training data randomly changed for each protein, HE  =  hold-out dataset error (%) i.e. error when predicting 30% of training data, OE  =  overall error (%) i.e. percentage of incorrect predictions, SN  =  sensitivity (%)  =  true positives/(true positives+false negatives), SP  =  specificity (%)  =  true negatives/(true negatives+false positives).

a Cross-validation involved a random sample of 70% from training dataset to build predictive model and remaining 30% used for testing. This was repeated 10 times and predictions averaged (predictions for the same input data fluctuate unless a random seed is set initially).

b Benchmark are proteins from published studies with known or expected T-cell responses (source species: *T. gondii*) –100% from training data used to build predictive model.

Note: Number of input variables used to build predictive model  = 304 (i.e. number of allele-peptide length combinations derived from 76 common alleles).

Using *randomforest* and *predict* has the advantage that it outputs an estimated probability for each protein of belonging to ‘YES’ and ‘NO’ classes e.g. for UniProt ID ‘A4GWX7’, 19.6% for ‘NO’ and 80.4% for ‘YES’ class. These probabilities can be used to rank the candidates. The probabilities in effect encapsulate all peptide-MHC binding scores from a protein and represent the predicted vaccine candidacy potential. A caveat here is that a protein assigned a high ‘YES’ probability does not necessarily imply a high probability of an immune response when injected in a host. However, it is assumed a high probability protein is more likely to contain the appropriate binding peptides to the restricted set of host MHC alleles than one with a lower probability.

### Results from amino acid conservation classification

**Figure 4:**
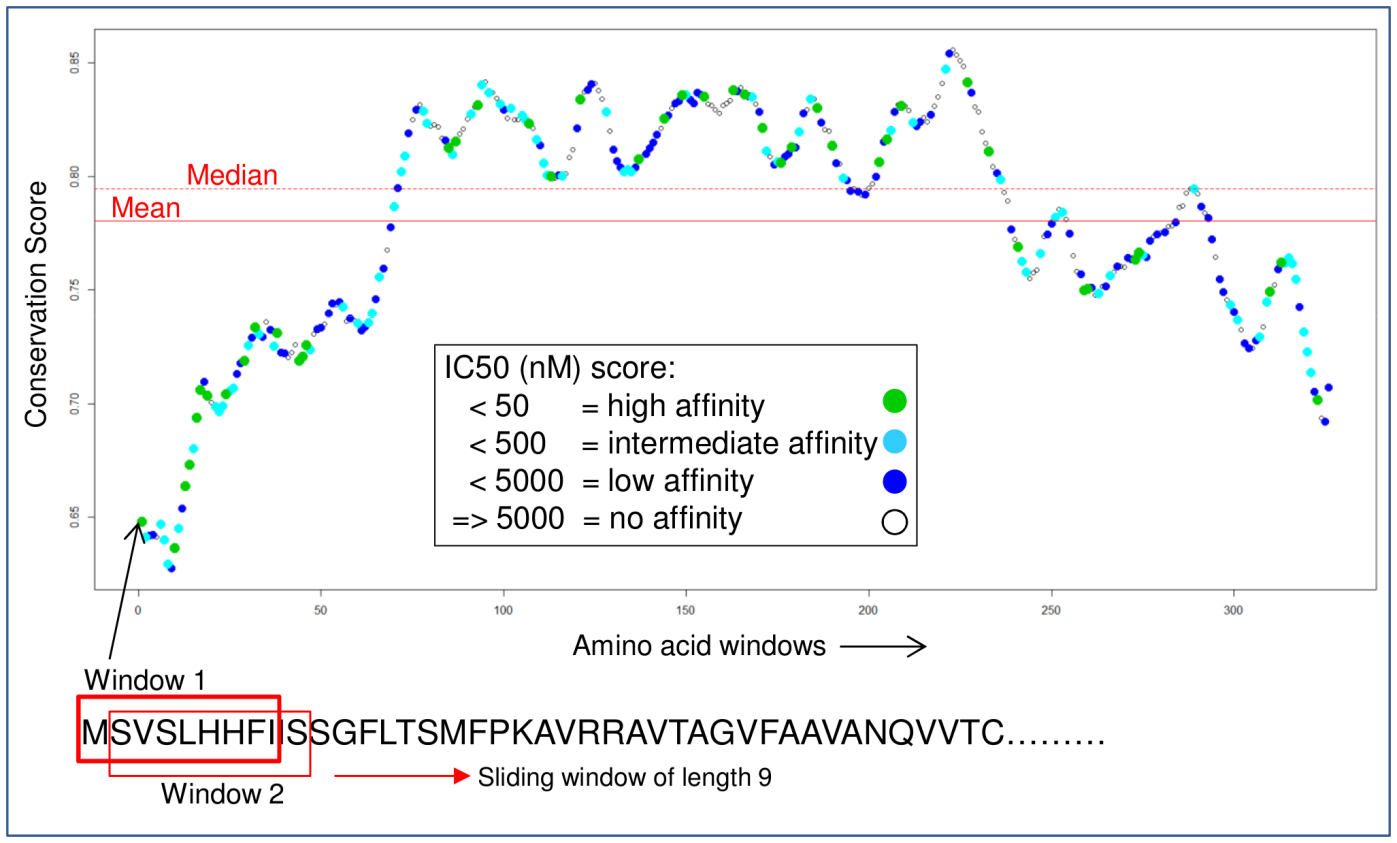
Plot of conservation scores computed for binding peptides along a protein (UniProtKB ID: P13664). Each circle represents the amino acid conservation score computed at a sliding window. The window is of length 9 and slides one residue at a time. The colour of the circle represents binding affinities against 76 common MHC alleles computed at each window. A window (i.e. a peptide) can theoretically bind to all 76 alleles and colours are therefore plotted in a set order: no, low, intermediate, and high affinity. For example, a dark blue circle for low affinity indicates there are no intermediate or high affinity peptides at the window; however, a green circle for high affinity provides no indication of other affinities at the same window. Mean conservation  = 0.7805; median conservation  = 0.7946. For protein P13664 (Major surface antigen p30) 54.6% high, 56% intermediate, and 55.9% low binders have conservation scores below the mean. The study shows that vaccine candidates are significantly more likely to have either a greater number of less conserved peptides or a lower total conservation score than non-vaccine candidates.

It is arguable whether the proposed ML strategy is only indirectly predicting secreted and membrane-associated proteins owing to the nature of the training data. Several secreted and membrane-associated proteins with no known immunogenicity history were also tested with the ML strategy. The challenge is that there is no way of validating, other than testing in a wet laboratory, if proteins with predicted low probabilities are truly non-vaccine candidates. The second proposed strategy, using amino acid conservation scores, requires no training data and independently supplements the ML strategy. Fig. 4 shows a plot of the conservation scores computed for a sliding window of nine amino acids in length along a protein. A window in this instance represents the length of a peptide. The general shape of the plot is typical for all proteins whereby some regions are more conserved than others. A binding score at each window/peptide against the 76 common alleles was predicted. Binding peptides with varying affinity strengths were typically found along the entire length in all tested proteins, as is the case in Fig. 4. The binding peptide distribution is also in keeping with the expected biology. That is, epitopes are expected to naturally occur in both conserved and non-conserved regions. The aim of the second strategy was to correctly classify each benchmark protein given only its collection of binding and associated conservation scores. Three techniques (described later in Materials and Methods) were used with varying degrees of success. The classification results were: SN  = 74.6% and SP  = 70.5% (when using mean difference as a threshold); SN  = 70.7% and SP  = 72.9% (with mean threshold); and SN  = 66.7% and SP  = 64.7% (with cumulative counts). All three techniques use a threshold for the binary classification that can be adjusted giving a trade-off between the two performance measures. Moreover, as with any threshold technique, prediction values close to the threshold warrant further evidence to support the classification. Despite the fluctuating results, the important finding is that there is a statistically significant relationship between a potential vaccine candidate and the conservation of its amino acids. That is, a vaccine candidate is significantly more likely to have either a greater number of less conserved peptides and/or a lower total conservation score than a non-vaccine candidate (Pearson’s Chi-squared test with Yates’ continuity correction: p-value <0.001). To further eliminate the possibility that the results were achieved by chance, the conservation scores were randomly shuffled and associated with a different binding score. That is, the average mean conservation for the protein remained the same but the association between the binding and conservation scores were random. In this instance, the classification results using mean difference as a threshold were: SN  = 52.7% and SP  = 47.9% (p-value  = 1).

### Comparison to other high-throughput prediction programs

**Figure 5:**
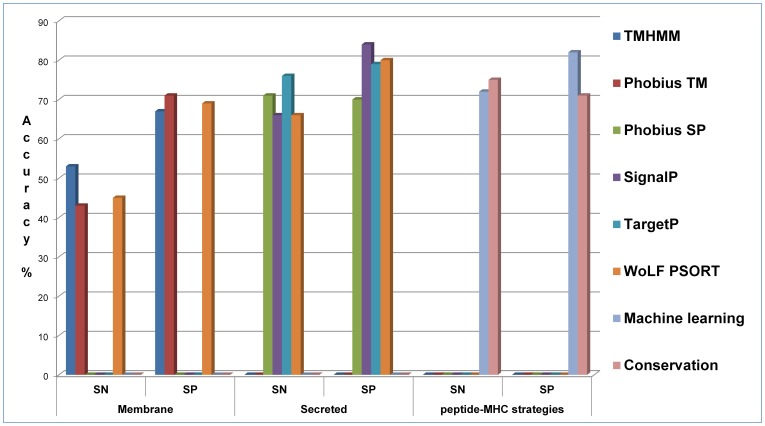
Performance comparison between high-throughput subcellular location predictors and peptide-MHC binding strategies. A column chart showing the sensitivity (SN) and the specificity (SP) performance measures for high-throughput programs in classifying 160 benchmark proteins as either membrane-associated or secreted. Predictors for membrane  =  TMHMM, Phobius TM, and WoLF PSORT; predictors for secreted  =  Phobius SP, SignalP, TargetP, and WoLF PSORT. Threshold criteria applied to each program’s specific output to achieve binary classification: TMHMM – membrane if tmhmm_ExpAA >18^$$^; Phobius TM – membrane if number of transmembrane domains >0; Phobius SP – secreted if value  =  ‘Y’; SignalP – secreted if SignalP_D >0.5; TargetP – secreted if value >0.5; WoLF PSORT – membrane if score >16^$$^ and annotation  =  ‘membrane’, or secreted if score >16^$$^ and annotation  =  ‘secreted’ (where ^$$^ is a value recommended by the creator of the program). Machine learning  =  strategy using random forest algorithm with peptide-MHC binding scores. Conservation  =  strategy using amino acid conservation of predicted binding peptides. Performance measures for peptide-MHC strategies are derived from classification of benchmark proteins as either vaccine or non-vaccine candidates.

There are alternative programs to the proposed peptide-MHC strategies as the framework for what constitutes a vaccine candidate in this study is essentially based on subcellular location. Five high-throughput programs (WoLF PSORT [47], SignalP [48], TargetP [49], TMHMM [50], and Phobius [51]) were used with protein sequences from the benchmark proteins as input. These programs predict protein characteristics relevant to subcellular location but in particular provide computationally evidence a protein is secreted or membrane-associated i.e. they can support or oppose a protein as a vaccine candidate. Fig. 5 shows a column chart as a comparison of the programs’ performances in classifying membrane-associated or secreted (S3 Supporting Information contains the exact predicted values). No program on its own provides sufficient evidence to draw conclusions for vaccine candidacy. Notwithstanding the inaccuracies of the programs, only WoLF PSORT can predict both membrane and secreted proteins. The presented strategies are also compared in Fig. 5. They compare favourably in distinguishing between immune exposed/unexposed proteins but do not differentiate the subcellular location. The crucial point nevertheless is that only some of the proteins predicted by the five programs will contain appropriate binding peptides to the restricted set of host MHC alleles. The peptide-MHC strategies can help determine these proteins. They are therefore not intended to compete but complement other evidence gathering programs. It is expected that an informed consensus towards candidacy will always be derived from multiple sources of evidence.

The core of the strategies is protein sequences. It is possible that linear sequences simply lack sufficient information to precisely predict immunogenicity. Peptide-MHC complexes and T-cell receptors are dynamic three-dimensional (3D) structures such that structure-based information is expected to more likely reveal signals for immunogenicity. These signals theoretically can be predicted. However, a virtual absence of apicomplexan structural data presently rules out a structure-driven approach. The proposed strategies provide a worthwhile interim approach until 3D data and appropriate new prediction strategies become readily available.

## Conclusion

The aim of this study was to determine whether predicted peptide-MHC I binding scores for thousands of proteins from a target pathogen could contribute evidence to the *in silico* discovery of vaccine candidates. Currently there is no published high-throughput approach for utilising such scores. An initial challenge to the study was that there is no clear consensus as to what type of protein constitutes an ideal vaccine candidate for a T-cell mediated response. Consequently, this study defined a likely vaccine candidate as a protein that is naturally exposed to the immune system *and* contains binding peptides to common host MHC alleles; and conversely, a non-vaccine candidate as an unexposed protein.

The two presented peptide-MHC classification strategies can provide reasonable evidence in comparison to alternative approaches but are faced with several performance limiting sources of error. For example, an unknown percentage of input sequences are incorrect leading to reduced binding and conservation prediction accuracy. Similarly, an unknown percentage of annotation is incorrect leading to misclassification for both test and training data. Furthermore, the prediction programs used have their own inherent inaccuracies. The expectation is that performances will improve as the quality of data inevitably improves over time. However, the strategies, even with perfect programs and data, are unlikely to ever reach perfection given the unpredictability of the biology in question. That is, both immune-exposed and unexposed proteins are likely to contain high affinity binding peptides and/or many binding peptides and/or promiscuous binders and/or peptides that bind to promiscuous MHCs, and conserved and non-conserved peptides. There is no universal set of rules that can be applied directly to binding scores to classify candidates. The strategies therefore rely on exploiting subtle tendencies in the data to achieve their classification of candidates as there is yet no definitive feature to precisely separate them. For instance, vaccine candidates have a tendency to have more binding peptides with low conservation scores and/or lower total conservation scores and/or average stronger binding affinities than non-vaccine candidates. False candidates are expected since exposed proteins containing binding peptides with predominantly high conservation scores and/or average weak binding affinities do exist; and conversely, unexposed proteins exist that contain binding peptides with predominantly low conservation scores and/or average strong binding affinities. An important point in the strategies’ favour is that the alternative approaches discussed only predict immune-exposed or unexposed proteins and do not take peptide binding affinities into account. The alternative approaches still require a filtering strategy otherwise every predicted exposed protein would require laboratory validation. The proposed strategies can contribute filtering evidence to the overall *in silico* vaccine discovery pool of evidence to help identify those proteins more worthy of validation. This ultimately will save time and money by reducing the number of false candidates assigned for validation. Random forest especially provides evidence in the form of an estimated probability for ranking candidates.

## Materials and Methods

Six groups of tests were performed: 1) Accuracy test on peptide-MHC binding predictor, 2) MHC class I binding test on the entire known proteome of *T. gondii,* 3) rule-based test, 4) comparison test with existing high-throughput programs that can indicate a protein’s subcellular location, 5) machine learning test, and 6) binding and conservation score test. Tests three to six are primarily for vaccine candidate classification. All tests that required binding scores used a Linux standalone version of the IEDB MHC I binding tools downloaded from http://tools.immuneepitope.org/main/html/download.html. Consensus [52], [53] was the chosen prediction method, which is the recommendation by the program providers. This method consecutively uses several prediction methods. For example, for each MHC allele and peptide length combination, the artificial neural network (ANN) method [54] is tried first, stabilized matrix method (SMM) [55] is tried next, and then scoring matrices derived from combinatorial peptide libraries (Comblib_Sidney2008) [56], and finally NetMHCpan [57] is tried if no previous method was available for the allele-length combination.

### Accuracy test on peptide-MHC binding predictor

The National Center for Biotechnology Information (NCBI) GI number for the parent proteins of 328 experimentally derived *T. gondii* peptides were obtained from the Immune Epitope Database (http://www.iedb.org/): 148 of these peptides were observed in published studies to bind to specific MHC I alleles with a measured affinity, and the remaining 180 peptides observed not to bind to MHC I alleles. The parent protein sequences were downloaded from NCBI for input to the peptide-MHC binding predictor. An in-house Perl script compared the *observed* affinity strength of each peptide to a particular MHC allele with the *predicted* affinity strength to the same allele.

### MHC class I binding test on the entire known proteome of *T. gondii*

The protein sequence for every *T. gondii* protein from the Universal Protein Resource knowledgebase (UniProtKB) was input into the peptide-MHC binding predictor. The purpose was to predict the peptides binding to the 76 common MHC I alleles (in effect, each protein was tested against 304 MHC allele-peptide length combinations). An in-house Perl script parsed the output to determine which proteins contained high-affinity peptides (i.e. IC_50_ (nM) binding score <50).

### Rule-based test for vaccine candidate classification

Protein sequences for 160 *T.gondii* proteins (75 positives and 85 negatives) were downloaded from UniProKB: 22 of the positive proteins have been observed to induce CMI responses and 48 have been experimentally shown to be membrane-associated or secreted; 11 have epitopes identified experimentally and some of these epitopes elicit significant humoral and cellular immune responses in vaccinated mice. The 85 negative proteins have UniProtKB annotated subcellular locations other than membrane-associated or secreted. All 160 proteins are referred to in the study as the benchmark dataset (see S2 Supporting Information).

The protein sequences were input into the peptide-MHC binding predictor to test for their binding affinity to the 76 common human MHC I alleles. This produced a binding score at each subsequence (i.e. a sliding window position along the sequence) of each protein and for each of the 304 MHC allele-peptide length combinations. More than one score can be output per subsequence when using the Consensus method.

Proteins were ordered and classified on: Lowest IC_50_ score per protein (threshold value  = 1.5), number of high-affinity peptides per protein (threshold value  = 200), number of different MHC alleles per protein that bound to high-affinity peptides (threshold value  = 74), maximum number of high-affinity peptides per protein that bound to a particular allele-peptide length combination e.g. protein Q6W3D0 was predicted to contain 58 high-affinity peptides that bound to the same allele (HLA-C*03∶03) with the same peptide length of 8 (threshold value  = 10), and total binding score per protein weighted for the length of protein (threshold value  = 32289). Another test involved grouping proteins that had high-affinity peptides that bound to the same MHC allele or allele-peptide length combination (i.e. promiscuous allele). This test was to determine if ‘YES’ candidates had preference to one particular allele and ‘NO’ candidates to another.

The same rules (i.e. threshold values) used for the benchmark dataset were applied to binding scores computed from 124 *P. falciparum* and 760 *C. elegans* protein sequences. These proteins were from a mixture of sub-cellular locations (half have UniProt annotation as secreted or membrane-associated and considered likely vaccine candidates; the other half have annotation related to nuclear and cytoplasmic locations and considered less likely vaccine candidates).

### Machine learning test for vaccine candidate classification

Three training datasets were created derived from: 1) 187 proteins from five different pathogenic species of the phylum Apicomplexa (*T. gondii, N. caninum*, *P. falciparum*, *Cryptosporidium parvum*, *Eimeria tenella*), 2) 760 proteins from *C. elegans*, and 3) 124 proteins from *P. falciparum*. Protein sequences were downloaded from UniProtKB. These sequences were consecutively input into the peptide-MHC binding predictor to generate binding scores against 304 allele-peptide length combinations per protein for each dataset. An in-house Perl script parsed the output and added the binding score (i.e. IC_50_ (nM) <5000) computed for each peptide-MHC length combination to obtain a total binding score per combination. The total score was divided by the length of the protein as there is a strong positive correlation between the total and length. An average score was computed if the output contained more than one score at each sliding window position. A separate file for each training dataset was compiled in a format as shown in Fig. 3. Note that other variations of the binding score statistics were tested as training data: lowest IC_50_ score for each peptide-MHC length combination, number of binding peptides per protein, number of different MHC alleles per protein binding to high-affinity peptides, and total binding score without length weighting. Proteins were selected based on subcellular location annotation in UniProtKB, although experimentally validated annotation is limited for *T. gondii*. Proteins from apicomplexan species that are known not to infect humans (e.g. *E. tenella*) were included to increase the number of training proteins. This seemed a reasonable approach, as the phylum Apicomplexa is monophyletic [58] and many of the biological processes and molecules possessed by *T. gondii* can also be found in other closely related species [19], [20]. Immune-exposed proteins were assigned ‘1’ as the target variable; unexposed assigned ‘0’.

The same benchmark dataset as per the rule-based test was used. However, the predicted binding scores for the 304 allele-peptide length combinations were compiled into the same format as Fig. 3, except no target variable column was required.

Two supervised machine learning algorithms were used via R functions from packages to build predictive classification models: random forest via the *randomForest* R function [59]; and support vector machines (SVM) via the *ksvm* R function [60], which is contained in the *kernlab* package. The best result for SVM was a sensitivity of 68.2%, specificity 73.1%, and an overall error rate of 29.6%. Random forest was chosen as the best method overall for solving this specific classification problem because it had an overall error rate of 22.5%. The *predict* R function with argument ‘type  =  “prob”’ was used to apply the models to the benchmark dataset. The argument instructs the output to have a class probability distribution at each terminal node (or leaf) rather than a single ‘YES’ or ‘NO’ class label. A threshold of greater than or equal to 0.5 for a vaccine candidate was used in the final classification of the benchmark proteins. The algorithm-specific R functions are described in S1 Supporting Information and pertinent arguments and intricacies are highlighted.

### Conservation and binding scores test for vaccine candidate classification

A standalone BLASTP [61] on NCBI nr database was used with the benchmark proteins as queries. BLAST downloaded from ftp://ftp.ncbi.nlm.nih.gov/blast/db/ncbi-blast-2.2.25+-ia32-linux.tar.gz and nr downloaded from ftp://ftp.ncbi.nlm.nih.gov/blast/db/. Homologous proteins with a 90% identity were selected from the BLASTP output. Amino acid conservation scores were computed for peptides of length nine to eleven for the entire length of each benchmark protein. For example, for a peptide length of 9, a conservation score was computed for 9 amino acids at a time sequentially moving one amino acid along the protein (i.e. a sliding window). This involved generating MSAs for each window/peptide with the homologous proteins and then computing an average conservation score for the peptide. Two standalone programs were used: clustal-omega (http://www.ebi.ac.uk/Tools/msa/clustalo/) for the MSAs and a conservation score program from http://compbio.cs.princeton.edu/conservation. Each peptide-MHC binding score for the 304 MHC allele-peptide length combinations were associated with the appropriate conservation score. To help visualise the data refer to Fig. 4. Three different counting classification techniques were applied to the data with the aim to obtain the optimum number of true positives and negatives: 1) using a mean threshold – if the binding peptide had a conservation score below the protein’s mean conservation then the ‘YES’ tally was increased by one, otherwise the ‘NO’ tally was increased by one (the median, which was extremely similar in value to the mean, was also used as a threshold but gave slightly poorer results); 2) using a mean difference threshold – the difference between the peptide and the mean conservation scores were accumulated for each binding peptide along the protein. The total difference below the mean was subtracted by the total difference above. A positive result indicates a ‘YES’ (see the Mean_Diff column in S3 Supporting Information); and 3) cumulative counts of the binding peptide conservation scores per protein – proteins were ordered in ascending cumulative counts i.e. from least to the most conserved. The length of the protein is a factor as there is a positive correlation between the number of binding peptides and length of the protein. The cumulative count was divided by the length of the protein prior to ordering. The median cumulative count was used as the threshold for the binary classification i.e. ‘YES’ if below and ‘NO’ if above threshold. Note that homologous proteins with a 50% identity were also tested but provided less accurate classifications. Also, some benchmark proteins had as few as three homologous proteins when using 90% identity. Incorrect choices or an inappropriate number of homologs could be a potential source for classification errors.

### Comparison prediction programs

Standalone Linux versions of the prediction programs used in this study were obtained from: http://wolfpsort.org/WoLFPSORT_package/version0.2/(WoLF PSORT); http://www.cbs.dtu.dk/services/SignalP/(SignalP); http://www.cbs.dtu.dk/services/TargetP/(TargetP); http://www.cbs.dtu.dk/services/TMHMM/(TMHMM); and http://phobius.binf.ku.dk/instructions.html (Phobius).

## Supporting Information

S1 Supporting Information **Provides background information on T-cell epitope prediction and machine learning algorithms used in the study.**
(PDF)

S2 Supporting Information **Contains a detailed compilation of proteins used in the benchmark dataset.**
(PDF)

S3 Supporting Information **Microsoft Excel spreadsheets containing results for the benchmark dataset.**
(XLSX)
