# Supplementary material for: Enhancing In Silico Protein-Based Vaccine Discovery for Eukaryotic Pathogens Using Predicted Peptide-MHC Binding and Peptide Conservation Scores
Source: PLoS One. 2014 Dec 29;9(12):e115745. doi: 10.1371/journal.pone.0115745 (PMC4278717; doi:10.1371/journal.pone.0115745)
Supplement: S1 Supporting Information — Provides background information on T-cell epitope prediction and machine learning algorithms used in the study. (PDF) [file pone.0115745.s001.pdf]

Contents

1 Introduction ... 1
2 Epitope prediction ... 1
2.1 Prediction methods used for MHC class I ... 4
3 Algorithms used for machine learning ... 5
4 References ... 10

1 Introduction

This document supplements the paper, '**Enhancing *in silico* protein-based vaccine discovery for eukaryotic pathogens using predicted peptide-MHC binding and peptide conservation scores**'. It provides background information on epitope prediction but in particular, peptide-MHC I allele binding predictions. The two main methods for peptide-MHC predictions that are used in the machine learning (ML) strategy presented in the paper are artificial neural network (ANN) and Stabilized matrix method (SMM). This document describes both methods. The last section is a description of two machine leaning algorithms, and in particular random forest, which are used to classify vaccines based on binding affinity scores.

2 Epitope prediction

The current trend in vaccine development is epitope-based due to its potential to be more specific, safer, and easier to produce than traditional vaccines [1]. At the time of writing, there were no commercial epitope-based vaccines. The literature, however, provides examples of the potential of epitope-based vaccines to be immunogenic and protective. Some examples under evaluation are: a human CD8(+) T-cell epitope-based vaccine against primary ocular herpes infection and disease [2]; a conserved HIV epitope that neutralizes genetically divergent HIV strains [3]; and an epitope-based vaccine capable of eliciting immune responses against *Schistosoma japonicum* [4]. There are of two types of epitopes on protein antigens: linear (or continuous) and conformational (or discontinuous). Linear epitopes are continuous stretches of amino acids in a protein sequence, and conformational epitopes are formed through folding of the protein bringing together discontinuous amino acid sequences to form the epitope [5]. Epitopes that are recognised by T-cells are called T-cell epitopes, and likewise, epitopes recognised by B-cells are B-cell epitopes. B cell epitopes contain both continuous (~10%) and discontinuous (~90%) epitopes, while the majority of T cell epitopes are continuous [5]. It is the recognition of epitopes on pathogens by T- and B-cells (and soluble antibodies) that activates the cellular and humoral immune response [6]. Several studies have shown that protective immunity to apicomplexans under study, and in particular *T. gondii* and *N.caninum*, is through cell-mediated responses which are regulated by the production of interferon γ [7-10].

Many methods have and are still being developed to computationally predict epitopes [1,5,11-14]. There are both sequence- and structure-driven [15] methods i.e. prediction based on amino acid sequences (primary structure) as opposed to three dimensional structures.

T-cell epitopes, which are typically short linear peptides, are created either as a result of proteasomal cleavage from antigenic proteins or from protein splicing [16]. Theoretically, there are two computational approaches to T-cell epitope prediction: direct and indirect. A direct approach involves predicting short peptides recognised by T-cells i.e. predicting epitopes based on the hypothesis that epitopes have amphipathic[1] structures [17]. An indirect method focuses on prediction of peptide binding to major histocompatibility complex (MHC) molecules. The MHC binds short peptides (i.e. epitopes) derived from host and pathogen proteins and presents them on cell surface for inspection by T-cell receptors (TCRs) in the surface of cytotoxic T lymphocytes (CTL) and helper T lymphocytes (HTLs). The CTLs recognise and kill infected or malfunctioning cells that present epitopes [11]. Different MHC molecules have different binding affinities for antigenic peptides i.e. each MHC allele binds to a limited set of peptides [12]. There are two main classes of MHC molecules: class I and class II. One important difference between the two classes is that the binding groove for MHC class I is *closed* at both ends and *open* at both ends for class II. [18] The open-ended MHC II molecule allows the bound peptide to have significant protrusions at both ends, which complicates the MHC II binding prediction [12]. As a consequence of the extension beyond the groove, MHC class II molecules tend to be of variable length but are typically 12 to 25 amino acids long [19]. Peptides that bind to MHC class I molecules are typically 8 to 11 amino acids long [20].

Indirect prediction is dependent on adequate MHC allele data from the host of the target pathogen. i.e. successful computational prediction requires training data with a sufficiently large set of MHC-peptide binding affinities that are experimentally validated [18]. Data for human MHC alleles, referred to as human leukocyte antigen (HLA), is by far the most abundant. Binding studies show that HLAs are the most polymorphic human genes known [21] and each HLA allele recognizes a restricted set of peptides [13]. Direct methods, as to date, have proved to be of insufficient accuracy [22] and this may be why the majority of T-cell epitope predictors currently found online are indirect methods. Table S1-1 lists some of the T-cell epitope predictors. The program ctlpred is the only direct method available online[2].

Only NetMHC, netMHCII, and tools from the Immune Epitope Database and Analysis Resource (IEDB) from Table S1-1 have standalone packages that can be downloaded. In particular, IEDB provides a download Linux package (for a 32 bit system) that contains a collection of peptide binding prediction tools for MHC class I and class II molecules. Included in the package are NetMHCpan and NetMHCIIpan, which are extended versions to NetMHC and netMHCII. The collection of tools is a mixture of Python scripts and Linux specific binary files. Python 2.5 or higher is therefore a prerequisite to run the tools. These tools take as input an amino acid sequence (or a set of sequences) and determine each subsequence's ability to bind to a specific MHC molecule. For MHC class I the available prediction methods are: artificial neural network (ANN) [23], Average relative binding (ARB) [24], Stabilized matrix method (SMM) [25], SMM with a Peptide-MHC Binding Energy Covariance matrix (SMMPMBEC), Scoring Matrices derived from Combinatorial Peptide Libraries (Comblib_Sidney2008) [26], Consensus [27], and NetMHCpan [28].

[1] a hydrophobic side facing the major histocompatibility complex molecule and a hydrophilic side interacting with the T-cell receptor

[2] Direct method program ctlpred at http://www.imtech.res.in/raghava/ctlpred/

Table S1-1. MHC binding peptide predictors

Name	MHC Class	Algorithm	URL (last viewed April 2013)
ProPred I	I	QM	http://www.imtech.res.in/raghava/propred1/index.html
MAPPP	I	Motif-based	http://www.mpiib-berlin.mpg.de/MAPPP/binding.html
NetMHC	I	ANN	http://www.cbs.dtu.dk/services/NetMHC/
PREDEP	I	Threading	http://margalit.huji.ac.il/Teppred/mhc-bind/index.html
nHLAPred	I	ANN	http://www.imtech.res.in/raghava/nhlapred/
EpiJen	I	Multistep algorithm	http://www.ddg-pharmfac.net/epijen/EpiJen/EpiJen.htm
HLABIND	I	Threading	http://atom.research.microsoft.com/hlabinding/hlabinding.aspx
PEPVAC	I	User profile, PSSM	http://imed.med.ucm.es/PEPVAC/
KISS	I	SVM	http://cbio.ensmp.fr/kiss/
MHC-Thread	II	Threading	http://www.csd.abdn.ac.uk/~gjlk/MHC-Thread/
ProPred	II	QM	http://www.imtech.res.in/raghava/propred/
MHC2Pred	II	SVM	http://www.imtech.res.in/raghava/mhc2pred/index.html
netMHCII	II	SMM-QM	http://www.cbs.dtu.dk/services/NetMHCII/
SVMHC	I & II	SVM	http://abi.inf.uni-tuebingen.de/Services/SVMHC
Rankpep	I & II	Profiles, PSSM	http://immunax.dfci.harvard.edu/Tools/rankpep.html
SYFPEITHI	I & II	Motif matrices	http://www.syfpeithi.de/home.htm
SVRMHC	I & II	SVM-regression	http://svrmhc.biolead.org/
EPIMHC	I & II	User Profiles	http://imed.med.ucm.es/epimhc/
POPI	I & II	SVM	http://iclab.life.nctu.edu.tw/POPI/
TEpredict	I & II	QM	http://tepredict.sourceforge.net/index.html
IEDB	I & II	Various	http://tools.immuneepitope.org/main/html/tcell_tools.html
MHCBench[++]	I & II	Evaluation	http://www.imtech.res.in/raghava/mhcbench/

[++] Used to evaluate MHC binding peptide prediction algorithms

Abbreviations: QM = quantitative matrices, ANN = artificial neural networks, PSSM = position-specific scoring matrix, SMM = stabilized matrix method, SVM = support vector machine.

A large scale evaluation of three MHC class I binding prediction methods (ANN, SMM, and ARB) was conducted in 2006 [29]. Each of the three methods predicts the quantitative affinity of a peptide for an MHC molecule. In the evaluation, the predicted affinities of all three methods were compared to a collection of experimentally measured peptide affinities to MHC class I molecules. Linear correlation coefficients were calculated between predicted and measured affinities on a logarithmic scale. The evaluation reported that ANN performed the best in a statistically significant manner (with a correlation coefficient of 0.69), followed by SMM (0.62) and then ARB (0.55) [29].

The performance of a prediction method is governed by the availability of MHC alleles. In other words, not all methods can currently make predictions for all MHC allele and peptide length combinations (e.g. there may be insufficient experimental data available to generate the combinations). The Consensus method is recommended by the creators because this method consecutively uses several prediction methods. For example, for each MHC allele and peptide length combination ANN method is tried first, SMM is tried next, and then comblib_Sidney2008, ARB, and finally NetMHCpan is tried if no previous method was available for the allele-length combination.

Prediction methods are encapsulated in two programs: predict_binding for MHC class I and mhc_II_binding for MHC class II. The method to use in the prediction is given as a command-line parameter. Figure S1-1 shows an example of the command line syntax. Only one MHC allele is analysed at a time. Therefore, the relevant program needs to be executed multiple times for each possible MHC allele-peptide length combination.

The MHC source species available in IEDB are: human, cow, pig, mouse, gorilla, chimpanzee, and macaque; and the peptide lengths that can be specified are typically from 8 to 11. A distinct set of MHC alleles and peptide lengths are related to each MHC source species.

```
## Command-line syntax For MHC I ##
predict_binding.py arb "HLA-A*02:01" 9 sequence.fasta
        # arb = ARB prediction method
        # "HLA-A*02:01" = MHC allele name
        # 9 = peptide length
        # sequence.fasta = name of file containing amino acid sequences in a FASTA format
## Command-line syntax For MHC II ##
mhc_II_binding.py consensus3 HLA-DRB1*03:01 sequence.fasta
        # consensus = consensus prediction method
        # HLA-DRB1*03:01 = MHC allele name
        # sequence.fasta = name of file containing amino acid sequences in a FASTA format
```

Figure S1-1. Command-line syntax for executing IEDB MHC binding peptide

Figure S1-2 shows a typical output from the MHC class I predictor using a Consensus method (some columns have been deleted and the format adjusted to fit output on the page). Beginning at the start amino acid (numbered 1) of each sequence (denoted by #), a test subsequence of a specific peptide length (e.g. PepLengh = 9) is created (e.g. Sequence = MSMEGDRPS and is located from amino acids 1 to 9 on sequence input #1). The subsequence is scored (e.g. in units of IC_{50}nM) for binding affinity against the MHC allele e.g. HLA-A*02:05, using different prediction methods i.e. scores are calculated for each amino acid at each position in the subsequence, which are then added to yield the overall binding affinity. In the example in Figure S1-2, method NetMHCpan was used because no previous method was available for the allele-length combination. However, the output could in theory contain scores from multiple methods if the method was available for the allele-length combination. The next test subsequence in Figure S1-2 is “SMEGDRPSG” from amino acids 2 to 10 on sequence input #1 and is scored against the same MHC allele, and so on. The affinity of the MHC allele and subsequence binding is greater the lower the IC_{50} value. The program creators propose a rough guideline for interpretation: peptides with IC_{50} values <50 nM are considered high affinity, <500 nM intermediate affinity, and <5000 nM low affinity.

Allele	#	Start	End	PepLength	Sequence	Method	IC50(nM)
HLA-A*02:05	1	1	9	9	MSMEGDRPS	NetMHCpan	6829.04
HLA-A*02:05	1	2	10	9	SMEGDRPSG	NetMHCpan	26123.53
HLA-A*02:05	1	3	11	9	MEGDRPSGA	NetMHCpan	3.32

Figure S1-2. Typical output from IEDB MHC I peptide binding predictor

2.1 Prediction methods used for MHC class I

Two methods in particular are used in the paper: artificial neural network (ANN) and Stabilized matrix method (SMM). Both are trained to perform quantitative predictions of peptide-MHC binding as opposed to predicting binding versus nonbinding.

The ANN method combines two types of neural network predictions. In half the networks the amino acid sequence is encoded using a conventional sparse (orthogonal) encoding and in the other half of the networks the amino acids are encoded as their Blosum50 scores to the 20 different amino acids [23]. Blosum *(BLOck SUbstitution Matrix)* is a substitution matrix used in sequence alignments of proteins. A Blosum score describes the rate at which one amino acid in a sequence changes to another over time. Substituting an amino acid with similar physicochemical properties is more likely to have a smaller impact on the structure and function of a protein than a substitution with an amino acid with dissimilar physicochemical properties. Blosum50, in this

particular case, uses a 50% identify threshold to address more distantly related proteins in computing the score. The final prediction is calculated as a linear combination of the two network predictions. Nielsen and colleagues (the developers of ANN method) state that the extra predictive power over other methods can be attributed to the mutual information between positions in a motif. In simpler prediction tools it is assumed that the amino acids at each position along the peptide sequence contribute with a given binding energy, which can be independently added up to yield the overall binding energy of the peptide [23]. These predictions fail to recognize correlated effects where the binding affinity of a given amino acid at one position is influenced by amino acids at other positions in the peptide. Two adjacent amino acids may, for example, compete for the space in a pocket in the MHC molecule [23]. The ANN method takes into account these correlated effects using combination of neural networks defined by different encoding schemes such as Blosum matrices and hidden Markov model encoding, in addition to the conventional sparse encoding.

The Stabilized Matrix Method (SMM) 2005) calculates matrices from quantitative affinity data of peptides binding to MHC molecules. SMM incorporates a regularization parameter that suppresses the noise present in the training data that is inevitably present in all experimental measurements. This method also extracts information from upper and lower boundary values that define the range of experimental measurements, which in certain instances are no longer quantitatively accurate [25]. Another advantage of SMM is that experimental data from randomized peptide libraries can be combined with conventional peptides. This helps avoid selecting sequences for training data that introduce bias e.g. over- or under-representing residues at specific sequence positions [25].

The difference in predictive performance between ANN and that of the matrix-driven methods occurs mainly for high-affinity peptides, confirming that the signal of higher order sequence correlation is most strongly present with these peptides [23]. If higher order sequence correlations are expected to be the dominant influence on experimental outcomes, ANN is better suited than the SMM method (SMM can only incorporate pair correlations between positions of a sequence) [25].

3 Algorithms used for machine learning

A **random forest** (RF) algorithm was used via the *randonForest* R function [30]. The general idea behind a random forest algorithm is the combining of multiple unpruned decision trees (a forest of trees) into a single ensemble of models. Each tree has an equal weight in voting for the final collective decision and a majority vote wins (for regression, the average value over the ensemble of regression trees is the outcome).

A single decision tree uses a recursive partitioning approach whereby a tree is created by recursively splitting the dataset into subsets based on a test of an input variable value until the splitting adds no further value to the prediction i.e. a point at which the subset has all or almost all of the same value of the target variable. A constant is fitted to terminal nodes or leaves: the most probable class (e.g. YES' or 'NO') for classification trees and a mean value for regression trees. The general idea is to minimise training error in each leaf by the choice of predictors i.e. variables at split points. In effect a built decision tree represents a set of rules that can be used to predict classifications of new data with the same input variables. The random forest algorithm uses a similar ensemble approach to Adaptive boosting but the models are built simultaneously as opposed to one after another (Adaptive boosting is a meta-algorithm in the sense that it is used in conjunction with other ML algorithms, e.g.

decision trees, to build multiple models with the objective to perform better than any one single model). The 'random' aspect of the algorithm is the randomness in which it selects the input variables and the observations from the training dataset to build each individual decision tree. The function allows a user to vary either or both the number of decision trees *and* the number of variables to try at each split in the decision tree. In standard trees, each node is split using the best split among all variables. In a random forest, each node is split using the best among a subset of variables randomly chosen at that node.

Following preliminary testing using samples from the training datasets used in the ML strategy, 500 decision trees and 13 variables at each split were used throughout the RF testing. All 304 input variables were used to build the optimum RF model. If the target variable is a factor e.g. YES or NO, *randomForest* performs classification; if the target value is continuous e.g. 1.0 or 0.0, *randomForest* performs regression. However, arguments to randomForest ultimately determines the type of tree e.g. if 'Candidate' represents the target variable, '*randomForest* (as.factor(Candidate) …' is used for classification trees, and *randomForest* (Candidate …' for regression trees.

randonForest computes an out-of-bag (OOB) estimate of error rate. The error is an indication that when the resulting model is applied to new data, the classification predictions are expected to be in error by a similar amount. Predictions for the same input data fluctuate when using random forest. For the ML strategy, *randonForest* and *predict* R functions were therefore executed multiple times (e.g. 100 runs) and the predictions averaged. Two in-house R functions were created to run these functions a user-defined number of times: runPred and makePred (see Figure S1-3).

An advantage of random forest (and other ensemble algorithms) is that unimportant variables that have little relationship to the target variable do not greatly impact the accuracy of the resulting model. Another advantage is that the input data does not need to be normalised.

A **support vector machine** (SVM) algorithm was used via the *ksvm* R function [31], which is contained in the *kernlab* package. SVM is a non-linear classifier. Support vectors lie at the edge of an area in feature space which presents a boundary between two classes of observations e.g. vaccine and non-vaccine candidates. The general idea is to identify a straight line in feature space that separates the classes. New observations can then be classified depending on which side of the line it falls on [32]. There are potentially 304 input variables (see Figure S1-5) and the data is not distributed in such a way that it can be linearly separated. The package provides several kernels (e.g. Radial Basis 'Gaussian', polynomial, linear, hyperbolic tangent, Laplacian, Bessel, ANOVA RBF, and spline) that transform the data into high-dimensional feature space i.e. in effect create a gap between the two classes allowing linear separation by a hyperplane. There are also several model types (e.g. C, nu, and bound-constraint classifications) which determine the hyperplane. Two in-house R functions were created to run the SVM algorithm: runPred and makePred (see Figure S1-4). SVM only uses support vectors to build a model rather than the entire training dataset. This has the advantage that the size of the training set is not normally an issue and the model is not as susceptible to outliers.

```
runPred <- function (no_of_repeats)
{
        require(randomForest, quietly=TRUE)
        sink(file = "predictions.txt")
        increment <- 1

        while( increment <= no_of_repeats) {
                out <- makePred ()
                cat("Run:",increment,"\n")
                print (out)
                increment <- increment + 1
        }

        sink ()
}


makePred <- function ()
{
    #training data
    train <- 1:nrow (dataset)
    #test data
    prediction <- 1:nrow (candidates)

    #The following is the start of the code for the 304 input variables (for brevity only 4 are shown).
    #The 76 common alleles are listed in Figure S1-5
    input <- c("HLA.A.01.01.8","HLA.A.01.01.9","HLA.A.01.01.10","HLA.A.01.01.11",  …

    #Target variable
    target  <- "Candidate"

    rf <- randomForest(as.factor(Candidate) ~ .,
            data=dataset[train,c(input, target)],
             ntree=500,
            mtry=13,
            importance=TRUE,
             na.action=na.roughfix,
            replace=FALSE)

    # Make a prediction using the Random Forest model
    pr_class <- predict(rf, na.omit(candidates[prediction, c(input, target)]),type="prob")
    # Extract the relevant variables from the dataset.
    sdata <- subset(candidates[prediction,], select=c("ID","Candidate"))
    # Output the combined data.
    output <- cbind(sdata,pr_class)
}
```

Figure S1-3. ***runPred*** **and** ***makePred*** **R functions for random forest**

```
runPred <- function (no_of_repeats)
{
        sink(file = "predictions.txt")
        library (kernlab)
        increment <- 1
        while( increment <= no_of_repeats) {
                out <- makePred ()
                cat("Run:",increment,"\n")
                print (out)
                increment <- increment + 1
        }
        sink ()
}

makePred <- function ()
{
    test_numbers <- 1:nrow (candidates)
    TrainingDS <- new.env ()
    evalq ({
                data <- dataset

        #The following is the start of the code for the 304 input variables (for brevity only 4 are
        #shown). The 76 common MHC I alleles are listed in Figure S1-5
                input <-
                c("HLA.A.01.01.8","HLA.A.01.01.9","HLA.A.01.01.10","HLA.A.01.01.11", …
                target  <- "Candidate"
                train_numbers <- 1:nrow (data)
                form <- formula (Candidate ~ .)
        }, TrainingDS)

        TrainingSVM <- new.env (parent=TrainingDS)
        evalq ({
                model <- ksvm (form,
                        kernel = "rbfdot",
                        type = "C-svc",
                        data=dataset[train_numbers,c(input, target)],
                        prob.model=TRUE)
                }, TrainingSVM)

    # Make a prediction using the SVM model
    pr_class <- predict(TrainingSVM$model, na.omit (candidates[test_numbers, c(TrainingDS$input)],))
    # Extract the relevant variables from the dataset.
    subset_data <- subset(candidates[test_numbers,], select=c("ID","Candidate"))
    # Output the combined data.
    output <- cbind(subset_data,pr_class)
}
```

Figure S1-4. *runPred* and *makePred* R functions for Support Vector Machines

HLA-A*01:01	HLA-B*08:01	HLA-B*46:01	HLA-C*05:01
HLA-A*02:01	HLA-B*13:01	HLA-B*48:01	HLA-C*06:02
HLA-A*02:06	HLA-B*13:02	HLA-B*49:01	HLA-C*07:01
HLA-A*03:01	HLA-B*14:02	HLA-B*50:01	HLA-C*07:02
HLA-A*11:01	HLA-B*15:01	HLA-B*51:01	HLA-C*07:04
HLA-A*23:01	HLA-B*15:02	HLA-B*52:01	HLA-C*08:01
HLA-A*24:02	HLA-B*15:25	HLA-B*53:01	HLA-C*08:02
HLA-A*25:01	HLA-B*18:01	HLA-B*55:01	HLA-C*12:02
HLA-A*26:01	HLA-B*27:02	HLA-B*56:01	HLA-C*12:03
HLA-A*29:02	HLA-B*27:05	HLA-B*57:01	HLA-C*14:02
HLA-A*30:01	HLA-B*35:01	HLA-B*58:01	HLA-C*15:02
HLA-A*30:02	HLA-B*35:03	HLA-B*58:02	HLA-C*16:01
HLA-A*31:01	HLA-B*37:01	HLA-C*01:02	HLA-C*17:01
HLA-A*32:01	HLA-B*38:01	HLA-C*02:02	HLA-E*01:01
HLA-A*33:03	HLA-B*39:01	HLA-C*02:09	HLA-G*01:01
HLA-A*68:01	HLA-B*40:01	HLA-C*03:02	HLA-G*01:02
HLA-A*68:02	HLA-B*40:02	HLA-C*03:03	HLA-G*01:03
HLA-A*74:01	HLA-B*44:02	HLA-C*03:04	HLA-G*01:04
HLA-B*07:02	HLA-B*44:03	HLA-C*04:01	HLA-G*01:06

Figure S1-5. ***Common human MHC I alleles***

Lists 76 MHC I alleles that commonly occur in at least 1% of the human population (18 HLA-A, 32 HLA-B, 20 HLA-C, 1 HLA-E, 5 HLA-G). MHC I binds to peptides, typically eight to eleven amino acid residues in length. There are potentially 304 MHC allele-peptide length combinations (76 alleles * 4 peptide lengths).

4 References

1. Zhao B, Sakharkar K, Lim C, Kangueane P, Sakharkar M (2007) MHC-Peptide binding prediction for epitope based vaccine design. International Journal of Integrative Biology 1.
2. Chentoufi AA, Dasgupta G, Christensen ND, Hu J, Choudhury ZS, et al. (2010) A Novel HLA (HLA-A*0201) Transgenic Rabbit Model for Preclinical Evaluation of Human CD8(+) T Cell Epitope-Based Vaccines against Ocular Herpes. Journal of Immunology 184: 2561-2571.
3. Paul S, Planque S, Nishiyama Y, Escobar M, Hanson C (2010) Back to the future: covalent epitope-based HIV vaccine development. Expert Review of Vaccines 9: 1027-1043.
4. Wang X, Zhang L, Chi Y, Hoellwarth J, Zhou S, et al. (2010) The nature and combination of subunits used in epitope-based Schistosoma japonicum vaccine formulations affect their efficacy. Parasites & Vectors 3.
5. Yang X, Yu X (2009) An introduction to epitope prediction methods and software. Reviews in Medical Virology 19: 77-96.
6. Flower DR, Macdonald IK, Ramakrishnan K, Davies MN, Doytchinova IA (2010) Computer aided selection of candidate vaccine antigens. Immunome Research 6 Suppl 2: S1.
7. Denkers EY, Gazzinelli RT (1998) Regulation and function of T-cell-mediated immunity during Toxoplasma gondii infection. Clinical Microbiology Reviews 11: 569-+.
8. Dlugonska H (2008) Toxoplasma rhoptries: Unique secretory organelles and source of promising vaccine proteins for immunoprevention of toxoplasmosis. Journal of Biomedicine and Biotechnology.
9. Innes EA, Andrianarivo AG, Bjorkman C, Williams DJL, Conrad PA (2002) Immune responses to Neospora caninum and prospects for vaccination. Trends in Parasitology 18: 497-504.
10. Williams DJL, Trees AJ (2006) Protecting babies: vaccine strategies to prevent foetopathy in Neospora caninum-infected cattle. Parasite Immunology 28: 61-67.
11. Korber B, LaBute M, Yusim K (2006) Immunoinformatics comes of age. PLoS Computational Biology 2: 484-492.
12. Lundegaard C, Hoof I, Lund O, Nielsen M (2010) State of the art and challenges in sequence based T-cell epitope prediction. Immunome Research 6 Suppl 2: S3.
13. Tong JC, Tan TW, Ranganathan S (2007) Methods and protocols for prediction of immunogenic epitopes. Briefings in Bioinformatics 8: 96-108.
14. Tsurui R, Takahashi T (2007) Prediction of T-cell epitope. Journal of Pharmacological Sciences 105: 299-316.
15. Knapp B, Omasits U, Frantal S, Schreiner W (2009) A critical cross-validation of high throughput structural binding prediction methods for pMHC. Journal of Computer-Aided Molecular Design 23: 301-307.
16. Hanada K, Yewdell JW, Yang JC (2004) Immune recognition of a human renal cancer antigen through post-translational protein splicing. Nature 427: 252-256.
17. Vivona S, Gardy JL, Ramachandran S, Brinkman FSL, Raghava GPS, et al. (2008) Computer-aided biotechnology: from immuno-informatics to reverse vaccinology. Trends in biotechnology 26: 190-200.
18. Wang P, Sidney J, Dow C, Mothe B, Sette A, et al. (2008) A systematic assessment of MHC class II peptide binding predictions and evaluation of a consensus approach. PLoS Computational Biology 4: e1000048.
19. Jardetzky TS, Brown JH, Gorga JC, Stern LJ, Urban RG, et al. (1996) Crystallographic analysis of endogenous peptides associated with HLA-DR1 suggests a common, polyproline II-like conformation for bound peptides. Proceedings of the National Academy of Sciences of the United States of America 93: 734-738.
20. Rammensee HG, Friede T, Stevanovic S (1995) MCH ligands and peptide motifs - first listing. Immunogenetics 41: 178-228.
21. Williams TM (2001) Human leukocyte antigen gene polymorphism and the histocompatibility laboratory. Journal of Molecular Diagnostics 3: 98-104.
22. Deavin AJ, Auton TR, Greaney PJ (1996) Statistical comparison of established T-cell epitope predictors against a large database of human and murine antigens. Molecular Immunology 33: 145-155.
23. Nielsen M, Lundegaard C, Worning P, Lauemøller SL, Lamberth K, et al. (2003) Reliable prediction of T-cell epitopes using neural networks with novel sequence representations. Protein Science 12: 1007-1017.
24. Bui H-H, Sidney J, Peters B, Sathiamurthy M, Sinichi A, et al. (2005) Automated generation and evaluation of specific MHC binding predictive tools: ARB matrix applications. Immunogenetics 57: 304-314.
25. Peters B, Sette A (2005) Generating quantitative models describing the sequence specificity of biological processes with the stabilized matrix method. BMC Bioinformatics 6: 132.
26. Sidney J, Assarsson E, Moore C, Ngo S, Pinilla C, et al. (2008) Quantitative peptide binding motifs for 19 human and mouse MHC class I molecules derived using positional scanning combinatorial peptide libraries. Immunome Research 4: 2.

27. Moutaftsi M, Peters B, Pasquetto V, Tscharke DC, Sidney J, et al. (2006) A consensus epitope prediction approach identifies the breadth of murine TCD8+-cell responses to vaccinia virus. Nature biotechnology 24: 817-819.
28. Hoof I, Peters B, Sidney J, Pedersen LE, Sette A, et al. (2009) NetMHCpan, a method for MHC class I binding prediction beyond humans. Immunogenetics 61: 1-13.
29. Peters B, Bui H-H, Frankild S, Nielsen M, Lundegaard C, et al. (2006) A community resource benchmarking predictions of peptide binding to MHC-I molecules. PLoS Computational Biology 2: 574-584.
30. Breiman L (2001) Random forests. Machine Learning 45: 5-32.
31. Platt J. Probabilistic Outputs for Support Vector Machines and Comparisons to Regularized Likelihood Methods; 1999. pp. 61-74.
32. Cortes C, Vapnik V (1995) Support-vector networks. Machine Learning 20: 273-297.
