# Supplementary material for: Enhancing In Silico Protein-Based Vaccine Discovery for Eukaryotic Pathogens Using Predicted Peptide-MHC Binding and Peptide Conservation Scores
Source: PLoS One. 2014 Dec 29;9(12):e115745. doi: 10.1371/journal.pone.0115745 (PMC4278717; doi:10.1371/journal.pone.0115745)
Supplement: S2 Supporting Information — Contains a detailed compilation of proteins used in the benchmark dataset. (PDF) [file pone.0115745.s002.pdf]

Contents

1 Introduction ... 1
2 Description of protein types from Table S2-1 ... 6
2.1 Membrane-associated proteins ... 6
2.2 Secreted proteins ... 7
2.3 Miscellaneous ... 8
3 Epitope and MHC binding evidence ... 9
4 REFERENCES ... 17

1 Introduction

This document supplements the paper, '**Enhancing *in silico* protein-based vaccine discovery for eukaryotic pathogens using predicted peptide-MHC binding and peptide conservation scores**'. It contains a compilation of *Toxoplasma gondii* proteins that are expected to make promising vaccine candidates. These proteins are used as the primary benchmark dataset to test proposed classification strategies presented in the paper.

There is currently no commercial subunit vaccine against Toxoplasmosis despite decades of research and millions of dollars spent [1]. However, the literature is full of examples of proteins observed to induce immune responses in animal models and therefore represent the type of proteins 'likely' to be worthwhile vaccine candidates. A level of caution is still warranted here because it is difficult to judge from published studies the expected efficacy of the candidates. We can only know for certain that candidates are truly worthwhile after testing in several target hosts. It is also difficult to make quantitative comparison of claimed protection levels in the studies because of differences in mouse models, ages of mice at the time-point of infection, vaccine delivery routes, adjuvants, vaccination and infection doses, parasite culture systems, challenge strains, and immunogenicity assessments [2]. For example, a candidate observed to poorly perform may give the desired protection with a different adjuvant or vaccine delivery route. Collectively, these studies indicate that the exact type and intensity of response and the immune correlates of protection are still unknown [2] but the typical type of candidate has a common theme. That is, the candidates are predominantly proteins that are naturally exposed to the immune system, such as membrane-associated proteins namely GPI-linked surface antigens (termed SAG), surface antigens known as SAG1-related sequences (SRS); and secreting proteins from specialized secretory organelles: micronemes produce MIC proteins that are involved in recognition of and adhesion to the host cell; rhoptries produce ROP proteins that drive the installation of the parasite inside the host cell; and dense granules produce GRA proteins that are involved in the maturation of the parasitophorous vacuole (PV) [3]. Three developmental stages of *T. gondii* (tachyzoite, bradyzoite and sporozoite) interact with the host immune system [1]. Therefore bradyzoite antigens (BAG) are also included in the compilation as likely candidates.

This document is in three parts: 1) Table S2-1, comprising the compiled proteins that are expected to be likely vaccine candidates with columns for Gene name, UniProt ID, Protein description, Epitope experimental evidence, Study publication reference, Machine learning (ML) classification probability for vaccine candidacy using random forest algorithm, Peptide conservation classification using predicted peptide-MHC binding and amino acid conservation scores, and Comments; 2) a brief description of some of the protein types listed in Table S2-1; and 3) Table S2-2 and S2-3, showing experimental epitopes and MHC binding information related to proteins in Table S2-1.

Some candidates in Table S2-1 have the words ‘no evidence’ instead of a study publication reference. This means that there is currently no publication to support the protein as a vaccine candidate. However, these proteins in question are included as likely candidates because they belong to gene families in which there are members that do have supporting immunogenic evidence. The gene families that encode *T. gondii* proteins [4] – SRS domain SAG1-like: SAG1, SAG1-related sequence 6, SAG1-related sequence 3, SAG3, SAG5D, BSR4, SAG5A, BSR4-related antigen; SRS domain SAG2-like: SAG2 (P22), SAG2B, SAG2C, SAG2D, SAG2E; Mucins and parlogs: GRA8, BAG Protein, GRA2; TRAP family: MIC2, MIC6, MIC8; EFG-like domain-containing: MIC3, MIC6, MIC7, MIC8; TSP1 domain-containing: MIC2; Apple domain-containing: MIC4; ROP nomenclature rhoptry proteins: ROP1, ROP2, ROP4, ROP5, ROP6, ROP8, ROP10, ROP12, ROP13, ROP14, ROP15, ROP16, RON1, RON2, RON3, RON4, Rhoptry neck protein 4-like protein; and GRA nomenclature dense-granule proteins: GRA1, GRA3, GRA4, GRA5, GRA6, GRA7 (p29), GRA8, GRA9, GRA10. A universal vaccine formulation is expected to ultimately be a cocktail of immunogenic proteins that occur in multiple strains and multiple life cycle stages. Suitable adjuvants are equally important components to the formulation.

A study to be highlighted here is by Che and colleagues [5]. The study involved a comprehensive proteomic analysis of membrane proteins in *T. gondii*. In brief, three proteomics strategies were used: one-dimensional gel electrophoresis liquid chromatography-tandem mass spectrometry (1D gel LC-MS/MS), biotin labelling in conjunction with 1D gel LC-MS/MS analysis, and a novel strategy that combined three-layer ‘Sandwich’ Gel Electrophoresis (TLSGE) with multidimensional protein identification technology (MudPIT) [5]. The transmembrane protein clusters identified in the study were deposited in the Einstein Biodefense Proteomics Research Center (http://toro.aecom.yu.edu/cgi-bin/biodefense/main.cgi) and the data provided to ToxoDB (http://ToxodB.org), which is part of EuPathDB. Only proteins identified by all three strategies and having one or more predicted transmembrane segments were included in Table S2-1. The experimental evidence for the epitope and MHC binding information in Table S2-3 and S2-3 was extracted from the Immune Epitope Database Analysis Resource (IEDB): http://www.iedb.org/.

Table S2-1. A list of proteins used in the benchmark dataset

Gene Name	UniProt ID	Protein Description	Epitope evidence	Study publication reference	ML classification Probability (%)	Peptide conservation classification	Comments
GRA1	P13403[1]	Dense granule protein 1	YES	[6,7]	79.3	YES	Signal peptide
GRA2	P13404[1]	Dense granule protein 2		[8]	83.2	YES	Signal peptide
GRA3	B6KEU8[1]	Dense granule protein 3	YES	[5,9,10]	79.1	NO	Contains 2 to 3 transmembrane helices + N-terminal secretory signal
GRA4	Q27002[1]	Dense granule protein 4	YES	[11]	80.8	NO	Signal peptide + transmembrane helices
GRA5	Q07828[1]	Dense granule protein 5	YES	[5,12]	76.4	YES	Signal peptide
GRA6	Q27003[1]	Dense granule protein 6	YES	[13-15]	83.2	NO	2 * transmembrane helices
GRA7	O00933[1]	Dense granule protein 7	YES	[13,14,16,17]	76.2	YES	Signal peptide + transmembrane helices + PTM.
GRA8	Q9U4T9	Dense granule protein 8		[5,18]	62.1	YES	100% ident with Q9GSE9 - P35 surface antigen
GRA9	B6KHS2	Dense granule protein 9		No evidence	40.0	YES	
GRA12	B5TSF4	Dense granule protein 12		No evidence	51.2	YES	
GRA14	B5TVE8	Dense granule protein 14		No evidence	63.3	YES	
GRA15	V4Z7A0	Dense granule protein 15		No evidence	66.7	YES	
ROP1	Q04151	Rhoptry protein		[19,20]			Signal peptide
	Q06AK3	Rhoptry antigen, putative (ROP2)		[11,21-23]			
ROP4	A7UDC8	Secretory rhoptry 4		[11,24]			
ROP5	A4L9V1	Rhoptry protein 5		[25]			
ROP5LA	I6YLU4	Rhoptry protein 5-like A		[25,26]	40.1	YES	
	Q5RZZ6	ROP6		No evidence	61.9	YES	
	B6E132	ROP7 protein		No evidence	47.1	YES	
ROP8	B9QMZ2	Rhoptry antigen		[5,27]	40.1	NO	Possibly a true negative
P36	Q9GV96	P36 protein (ROP9)		[28]	44.8	YES	
ROP10	A4GWX7	Rhoptry protein 10		No evidence	80.4	NO	
ROP11	M9UUR3	Rhoptry protein 11		No evidence	45.4	NO	
ROP12	Q45WB2	Rhoptry protein 12		No evidence	59.5	NO	
ROP13	Q45WB1	Rhoptry protein ROP13		[29]	47.5	YES	

Abbreviation: PTM = post-translational modification

[1] Protein manually annotated and reviewed in UniProtKB. All other proteins are automatically annotated and not reviewed in UniProtK.

[2] ORF name – a name temporarily attributed to an open reading frame (ORF) by a sequencing project.

Gene Name	UniProt ID	Protein Description	Epitope evidence	Study publication reference	ML classification Probability (%)	Peptide conservation classification	Comments
TGVEG_211290[2]	B9QHX6	Rhoptry protein ROP15		No evidence	37.5	NO	Possibly a true negative
ROP16	B9QAP5	Rhoptry protein ROP16		[17,30]	47.4	YES	
ROP17	B6KB88	Rhoptry protein ROP17	YES	[31]	41.2	NO	
ROP18	Q2PAY2	Rhoptry kinase family protein		[5,32-34]	29.0	YES	
RON1	Q45WA9	Rhoptry neck protein 1		[5,35-37]	82.4	NO	
RON2	B6KV60[1]	Rhoptry neck protein 2		[5,38]	40.0	YES	Transmembrane helix
RON2L2	B6KLP1[1]	Rhoptry neck protein 2-like protein 2		[39]	54.5	NO	
RON3	Q45WA7	Rhoptry neck protein 3		[5,35,36,40]	46.1	NO	
RON4	B6KJ32[1]	Rhoptry neck protein 4		[41]	80.1	NO	Signal peptide
RON5	V4ZS24	Rhoptry neck protein 5		[42,43]	22.7	NO	
TGGT1_306060[2]	S7UI99	Rhoptry neck protein 8		[5,35,36]			
RON11	M4PVL7	Rhoptry neck protein 11		[44]	30.0	NO	
MIC1	O00834[1]	Micronemal protein 1	YES	[5,45]	81.9	YES	Signal peptide
MIC3	V4YND8	Microneme protein MIC3		[46,47]	84.9	YES	Signal peptide
MIC4	Q9XZH7[1]	Micronemal protein 4		[48-50]	87.6	NO	Signal peptide
MIC5	P90611	Micronemal protein		No evidence	42.4	YES	
MIC6	Q9XYH7[1]	Micronemal protein 6		[51,52]	89.1	YES	Signal peptide + transmembrane helix
MIC7	V4Z9J7	Micronemal protein 7		No evidence	75.4	YES	
MIC8	D8UY22	Micronemal protein 8		[53-56]	84.6	YES	
MIC9	Q9BIM6	Micronemal protein 9		No evidence	80.8	YES	
MIC11	Q8IT73	Microneme protein TgMIC11		[23,57]	32.0	YES	
MIC13	B0LUH4	Microneme protein 13		[58,59]	76.9	NO	Signal peptide
MIC15	Q1PA41	Microneme protein 15		No evidence	36.7	YES	
	B3VQI5	Microneme protein 16		No evidence	58.4	YES	
	V4Z8E3	Microneme protein 17a		No evidence	57.7	YES	
PLP1	V5BCL0	Perforin-like-protein 1		[60]	60.9	YES	
NTP1	Q27895[1]	Nucleoside triphosphate hydrolase 2		[61]	78.5	NO	
TgIb.2380c	Q1JSB4	Gpi-anchored surface bsr4-related antigen		No evidence	64.5	YES	
TgIb.2350c	Q1JSB6	Gpi-anchored surface bsr4-related antigen		No evidence	61.0	YES	Signal peptide

Abbreviation: PTM = post-translational modification

[1] Protein manually annotated and reviewed in UniProtKB. All other proteins are automatically annotated and not reviewed in UniProtK.

[2] ORF name – a name temporarily attributed to an open reading frame (ORF) by a sequencing project.

Gene Name	UniProt ID	Protein Description	Epitope evidence	Study publication reference	ML classification Probability (%)	Peptide conservation classification	Comments
TgIa.0200	Q1JTJ4	Gpi-anchored surface bsr4-related antigen		No evidence	47.0	YES	
SAG1	P13664	SAG1 protein (P30)	YES	[11,22,62-65]	27.6	YES	Signal peptide
SRS2	O15695	SAG1-related sequence 2		[66-68]	54.8	YES	
SAG2	B6KD48	Surface antigen 2		[69-71]	62.3	YES	
SAG2	Q95NF7	Surface antigen P22		[69-71]	65.8	YES	
SRS-6	Q9NH12	SAG1-related sequence 6		[72]	63.2	YES	
TgIb.1210	Q9XYH0	SAG2 related antigen SAG2B		[73,74]	55.6	YES	
	Q9XYH1	SAG2 related antigen SAG2C		[73-75]	58.4	YES	
	E2IPQ4	SAG3 (P43)		[76]	50.5	YES	
SAG5B	O15728	Surface antigen 5B		[77]	66.6	YES	
SAG5D	Q6WAX5	SAG-related sequence SRS36B		[78]	63.0	YES	
TGVEG_321480[2]	B9QQK9	SAG-related sequence SRS12B		No evidence	69.5	YES	
TGVEG_320240[2]	V5B1T6	SAG-related sequence SRS15B		No evidence	62.8	YES	
TGVEG_320180[2]	V4Z799	SAG-related sequence SRS16C		No evidence	50.9	YES	
TGVEG_258550[2]	B9PL31	SAG-related sequence SRS28		No evidence	52.9	YES	
TGVEG_233450[2]	B9Q4G2	SAG-related sequence SRS29A		No evidence	69.5	YES	
TGVEG_280580[2]	B9QPN9	SAG-related sequence SRS35B		No evidence	53.4	YES	
TGVEG_440320[2]	V4ZJG5	SAG-related sequence SRS36C		No evidence	57.3	YES	
TGVEG_267140[2]	V5BDH5	SAG-related sequence SRS38B		No evidence	56.9	YES	
TGVEG_224790[2]	B9Q7L4	SAG-related sequence SRS40A		No evidence	50.5	NO	
TGVEG_224760[2]	B9Q7L3	SAG-related sequence SRS40E		No evidence	55.7	YES	
TGVEG_296640[2]	V4Z2B8	SAG-related sequence SRS48E		No evidence	59.9	YES	
TGGT1_207130[2]	S7VYK0	SAG-related sequence SRS49A		No evidence	51.2	YES	
BAG5	Q26999	Bradyzoite antigen		[79,80]	54.0	YES	
SAG4	P90612	Bradyzoite surface protein		[81]	51.0	YES	Signal peptide
pgam2	Q1KSE9	Phosphoglycerate mutase 2 (EC 5.4.2.1)		[82,83]	46.8	YES	100% identity with S7UKZ1 (pgam1)

Abbreviation: PTM = post-translational modification

[1] Protein manually annotated and reviewed in UniProtKB. All other proteins are automatically annotated and not reviewed in UniProtK.

[2] ORF name – a name temporarily attributed to an open reading frame (ORF) by a sequencing project.

2 Description of protein types from Table S2-1

Host cell invasion is the key element in the pathogenesis of Toxoplasmosis. A tachyzoite invades a host cell first by recognising host-cell surface receptors via by a family of highly abundant surface antigens (e.g. different glycoproteins, SAGs. SRSs) on its cell membrane, and then secreting proteins in a regulated and sequential progression from specialized secretory organelles in the apical complex [5]. Firstly, microneme proteins (MICs) are released to form an attachment between the apical tip of the parasite and the host cell plasma membrane (i.e. parasite-host cell adhesion). The initial attachment is randomly orientated and the tachyzoite repositions to attach its apical end against the host. Immunodominant surface antigens such SAG-related sequence 2 are involved in the attachment [84,85]. Apicomplexans are extremely polarised because secretion from the apical complex is required for invasion. Entry is through a restricted attachment called a moving junction that joins the parasite pellicle with the host. Secondly, proteins (ROPs) are secreted by rhoptries through the protruding conoid to initiate invagination of the host cell membrane. The tachyzoite penetrates by pulling the moving junction posteriorly along its pellicle. The formation of a parasitophorous vacuole (PV) membrane is created by the inversion of the host membrane as the tachyzoite penetrates in [86]. Then, proteins (GRAs) are secreted sequentially from the dense granule organelles after the parasite is fully within the PV. Mercier and colleagues provide a comprehensive review on dense granules in the context of *T. gondii* [87]. The dense granule proteins are abundantly expressed in the PV and recent data suggest that some of these proteins could be involved in building an intravacuolar membranous nanotubular network [87,88]. The exact function of this elaborate network remains unclear but is currently believed to be used to extract nutrients from the host cell. The parasite is able to shelter, grow and replicate, and utilize components from the host for its survival whilst enveloped within the PV. The release of proteins from the three secretory organelles in a sequential progression implies that their release is governed by separate signals defining distinct phases of intracellular parasitism. Upon invasion of host cells the parasite is able to partially evade the humoral and cell-mediated immune response by altering the expression and secretion of immunomodulatory cytokines or by altering the capability of immune cells. The parasite needs the survival of the host in order to develop. So in effect the parasite exists in a delicate balance of inducing and suppressing the host's immune response [89].

The following describe some of the important candidate protein types in more detail although in no particular order of importance, and are grouped into three sections: membrane-associated, secreted and miscellaneous.

2.1 Membrane-associated proteins

SAG1, SAG2, and SAG3 are tachyzoite glycosylphosphatidylinositol (GPI)-anchored surface molecules [90] involved in host cell attachment and invasion [91] and are antigenically immunodominant [67]. SAG1/P30 was shown to elicit both humoral and cellular immune response [92] and is therefore a leading candidate for vaccine development [93] but is tachyzoite stage-specific. Using liposomes as adjuvant, purified SAG1/P30 was shown to provide protection of mice from a fatal *T. gondii* infection [62]. In another study, immune splenocytes from mice immunized with P30 appeared to lyse peritoneal macrophages infected with *T. gondii* [64]. Mice primed with recombinant influenza virus and boosted with a recombinant adenovirus encoding SAG2 elicited

both humoral and cellular immune responses specific for SAG2 [71]. A compound DNA vaccine encoding *T. gondii* antigens SAG1, SAG3 with CTXA(2)/B gene was shown to effectively enhance the humoral and cellular immune response and prolong survival time in vaccinated mice [76].

The surface of *T. gondii* is coated with developmentally expressed, GPI-linked proteins structurally related to SAG1. Collectively, these surface antigens are known as the SRS (SAG1-related sequences) superfamily of proteins [94]. **SRS2** [85] is localised on both bradyzoites and tachyzoites [95]. The SRS2 protein is involved in the host cell invasion process [96] and polyclonal and monoclonal antibodies directed against it were shown to inhibit invasion of placental ovine trophoblasts *in vitro* [97]. **SRS domain containing proteins** are present in large numbers on the parasite surface and facilitate the invasion of multiple host and cell types [94]. They are considered to be extremely immunogenic in *Toxoplasma* [23].

RON proteins originate from the neck of the rhoptries. All RON proteins have been demonstrated to be present at the moving junction between the apex of Apicomplexa and the host cell membrane that moves along the parasite and serves as support to propel it inside the host cell [35]. The moving junction assembly is initiated by injection of RONs into the host cell, where RON2 spans the membrane and functions as a receptor for apical membrane antigen 1 (AMA1) on the parasite [38]. The proteomics Che study included them as members of transmembrane proteins. Interestingly, the five prediction programs in the paper typically indicate RONs as both membrane-associated and secreted.

2.2 Secreted proteins

GRA proteins are involved in the cellular invasion process. Dense granules are secretory vesicles that play a major role in the structural modifications of the parasitophorous vacuole (PV) in which the parasite develops [98].

Both humoral and cellular immune responses against *T. gondii* was detected in sheep immunized with DNA plasmids encoding *T. gondii* GRA7 formulated in an adjuvant formulation [99]. Studies using antibodies to immunolocalize the *T. gondii* dense granule protein GRA3 have shown that this protein associates strongly with the parasitophorous vacuole membrane (PVM) i.e. GRA3 has an N-terminal secretory signal sequence and a transmembrane domain consistent with its insertion into the PVM. GRA3 possesses a dilysine 'KKXX' endoplasmic reticulum (ER) retrieval motif that interacts with PVM and the calcium modulating ligand of host cell ER in the parasitism of *T. gondii* [9,10]. The five prediction programs indicate that GRA3, and most other dense granule proteins described here, are both membrane-associated and secreted. GRA2 and GRA4 are not predicted to be membrane-associated.

MIC proteins are discharged by exocytosis during the attachment to the host cell surface to facilitate cell invasion [100]. Many microneme proteins also contain well-conserved functional domains associated with mainly adhesive activity (e.g. EGF-like and PAN_1 domains) and some protease activity (e.g. Peptidase_S8 and Rhomboid) [101].

MIC3 is expressed in all three infectious stages of *T. gondii* (tachyzoites, bradyzoites, and sporozoites). A DNA vaccine encoding the MIC3 protein has been demonstrated to elicit a strong specific immune response providing significant protection against *T. gondii* infection [47].

ROP proteins are involved in a variety of cellular functions related to host cell invasion, formation of the parasitophorous vacuole, and parasite-host cell interplay [102]. The protein combinations of rROP2 + rROP4 +

rGRA4 and rROP2 + rROP4 + rSAG1 were shown to be very effective in the development of a high level of protection irrespective of the genetic backgrounds and innate resistance to toxoplasmosis of the laboratory mice [11]. A DNA vaccine encoding the ROP1 antigen of *T.gondii* and ovine CD154 was demonstrated to stimulate humoral and cellular immune responses in sheep. The intramuscular injection of pROP1 only induced a Th1-specific immune response [19]. ROP2/P64 expresses in all three life cycle stages. ROP2, is involved in invasion of host cells, induces humoral immune response [22,23].

2.3 Miscellaneous

The following proteins were included in the benchmark dataset because they were mentioned in published studies as possible vaccine candidates. The first notable fact about these proteins is that they are not expected to be naturally exposed to the immune system.

Phosphoglycerate mutase (pgam) was identified using mass spectrometry as one of three main proteins in the excretory secretory antigen (ESA) [83]. The other two proteins were MIC10 and GRA7. The study authors conclude that these three proteins demonstrated good immunogenicity and may potentially be useful in the development of vaccines against toxoplasmosis either used singly or in various combinations. The results shown in Supporting Information S3 suggest that pgam is neither secreted nor membrane-associated and there is no consensus for vaccine candidacy.

A study [60] demonstrated that protective humoral and cellular immunity against experimental toxoplasmosis in susceptible Kunming mice was induced by a DNA vaccine encoding the **perforin-like protein 1** (PLP1). Furthermore, another study [52] showed that the immune efficacy induced by DNA vaccine with MIC6 and PLP1 was better than that induced by PLP1 or MIC6 alone. The results shown in Supporting Information S3 suggest that PLP1 is neither secreted nor membrane-associated but there is consensus from the peptide-MHC binding strategies.

Nucleoside triphosphate hydrolase (NTPase) is released from dense granules and accumulates as a soluble protein in the vacuolar space [61]. A study [61] described that a recombinant form of NTPase co-administered with the adjuvant alum induced a strong specific Th1 immune response against toxoplasmosis in a murine model. The results shown in Supporting Information S3 suggest that NTP1 is a secreted protein but there is no consensus for vaccine candidacy.

3 Epitope and MHC binding evidence

Table S2-2. Experimentally validated T-cell epitopes related to benchmark proteins in Table S2-1

UniProt ID	Homolog UniProt ID (90% Identity)	Epitope ID	Sub-sequence	NCBI GI#	Source Molecule Name
A8I7P3	O00933	147936	LPQFATAAT	157824702	granule antigen protein GRA7
B5B4W9	Q07828	139759	GLAAAVVAV	195984531	dense granule antigen precursor
B5B4W9	Q07828	140659	AVVSLLRLLK	195984531	dense granule antigen precursor
B6KEU8		139746	FLVPFVVFL	308154338	Dense granule protein 3
B6KEU8		148397	VPFVVFLVA	308154338	Dense granule protein 3
D2Y4V7	Q27003	117913	HPGSVNEFDF	283580621	dense granule antigen protein 6
D2Y4V7		139747	FMGVLVNSL	283580621	dense granule antigen protein 6
D2Y4V7	Q27003	139947	VVFVVFMGV	283580621	dense granule antigen protein 6
D2Y4V7	Q27003	140651	AMLTAFFLR	283580621	dense granule antigen protein 6
P13403		10421	DTMKSMQRDED	129322	Dense granule protein 1 precursor
P13664		1161	AESKSVII	129348	Major surface antigen p30 precursor
P13664		27248	ILPKLTENPWQ	129348	Major surface antigen p30 precursor
P13664		63089	TCPDKKSTA	129348	Major surface antigen p30 precursor
P13664	P13664	65781	TPTENHFTL	129348	Major surface antigen p30 precursor
Q27002		58128	SGLTGVKDSSS	2498423	Dense granule protein 4 precursor

UniProt ID	Homolog UniProt ID (90% Identity)	Epitope ID	Sub-sequence	NCBI GI#	Source Molecule Name
Q27002		104255	SPMNGGYYM	2498423	Dense granule protein 4 precursor
Q27298	P13664	40286	LVCGKDGVK	37778533	SAG1 protein
Q27298	P13664	60031	SPEKHHCTV	37778533	SAG1 protein
Q27298		65118	TLVCGKDGV	37778533	SAG1 protein
Q27298		140697	KSFKDILPK	37778533	SAG1 protein
Q9BJ38		75280	YPESGPVNL	13447092	surface antigen
Q9BJ38		139742	FLLGLLVHV	13447092	surface antigen
S8F3P1	O00834	139938	VLLPVLFGV	237838467	microneme protein MIC1
S8F944	O00933	140739	RSFKDLLKK	237836631	dense granule protein 7
S8GBP7		139785	ITMGSLFFV	237830827	SRS domain-containing protein

Table S2-3. Experimentally validated peptide-MHC I binding related to benchmark proteins in Table S2-1

UniProt ID	NCBI GI	Homolog ID (90% identity)	Epitope ID	Sub-sequence	Molecule Source Name	MHC Allele Name	Host Organism Name	Pubmed ID
A8I7P3	157824702	O00933	140739	RSFKDLLKK	granule antigen protein GRA7	HLA-A*11:01	Homo sapiens	21129215
A8I7P3	157824702	O00933	147936	LPQFATAAT	granule antigen protein GRA7	HLA-B*07:02	Mus musculus HLA-B*0702 Tg	22027386
A8I7P3	157824702	O00933	147936	LPQFATAAT	granule antigen protein GRA7	HLA-B*07:02	Homo sapiens	22027386
A8I7P3	157824702	O00933	147936	LPQFATAAT	granule antigen protein GRA7	HLA-B*07:02	Mus musculus HLA-B*0702 Tg	22027386
A8I7P3	157824702	O00933	147936	LPQFATAAT	granule antigen protein GRA7	HLA-B*07:02	Mus musculus HLA-B*0702 Tg	22027386
A8I7P3	157824702	O00933	147936	LPQFATAAT	granule antigen protein GRA7	HLA-B*07:02	Mus musculus HLA-B*0702 Tg	24736000
A8I7P3	157824702	O00933	147936	LPQFATAAT	granule antigen protein GRA7	HLA-B*07:02	Mus musculus HLA-B*0702 Tg	24736000
A8I7P3	157824702	O00933	147936	LPQFATAAT	granule antigen protein GRA7	HLA-B*07:02	Mus musculus HLA-B*0702 Tg	24736000
B5B4W9	195984531	Q07828	139759	GLAAAVVAV	dense granule antigen precursor	HLA-A*02:01	Homo sapiens	21095258
B5B4W9	195984531	Q07828	139759	GLAAAVVAV	dense granule antigen precursor	HLA-A*02:02	Homo sapiens	21095258
B5B4W9	195984531	Q07828	139759	GLAAAVVAV	dense granule antigen precursor	HLA-A*02:03	Homo sapiens	21095258
B5B4W9	195984531	Q07828	139759	GLAAAVVAV	dense granule antigen precursor	HLA-A*02:06	Homo sapiens	21095258

UniProt ID	NCBI GI	Homolog ID (90% identity)	Epitope ID	Sub-sequence	Molecule Source Name	MHC Allele Name	Host Organism Name	Pubmed ID
B5B4W9	195984531	Q07828	139759	GLAAAVVAV	dense granule antigen precursor	HLA-A*68:02	Homo sapiens	21095258
B5B4W9	195984531	Q07828	139759	GLAAAVVAV	dense granule antigen precursor	HLA-A*02:01	Mus musculus HLA-A*0201/Kb Tg	21095258
B5B4W9	195984531	Q07828	140659	AVVSLLRLLK	dense granule antigen precursor	HLA-A3	Homo sapiens	21129215
B5B4W9	195984531	Q07828	140659	AVVSLLRLLK	dense granule antigen precursor	HLA-A*11:01	Mus musculus HLA-A*1101/Kb Tg	21129215
B6KEU8	308154338		139746	FLVPFVVFL	dense granule protein GRA3	HLA-A*02:01	Homo sapiens	21095258
B6KEU8	308154338		139746	FLVPFVVFL	dense granule protein GRA3	HLA-A*02:01	Mus musculus HLA-A*0201/Kb Tg	21095258
B6KEU8	308154338		139746	FLVPFVVFL	Dense granule protein 3	HLA-A*02:01	Homo sapiens	20347630
B6KEU8	308154338		139746	FLVPFVVFL	Dense granule protein 3	HLA-A*02:03	Homo sapiens	20347630
B6KEU8	308154338		139746	FLVPFVVFL	Dense granule protein 3	HLA-A*02:06	Homo sapiens	20347630
B6KEU8	308154338		139746	FLVPFVVFL	Dense granule protein 3	HLA-A*68:02	Homo sapiens	20347630
B6KEU8	308154338		139746	FLVPFVVFL	Dense granule protein 3	HLA-A*02:02	Homo sapiens	20347630
B6KEU8	308154338		148397	VPFVVFLVA	Dense granule protein 3	HLA-B*07:02	Homo sapiens	20347630
B6KEU8	308154338		148397	VPFVVFLVA	Dense granule protein 3	HLA-B*42:01	Homo sapiens	20347630
B6KEU8	308154338		148397	VPFVVFLVA	Dense granule protein 3	HLA-B*54:01	Homo sapiens	20347630
B6KEU8	308154338		148397	VPFVVFLVA	dense granule protein GRA3	HLA-B*07:02	Homo sapiens	22027386
D2Y4V7	283580621	Q27003	117913	HPGSVNEFDF	dense granule antigen protein 6	H-2-Ld	Mus musculus BALB/c	18587399

UniProt ID	NCBI GI	Homolog ID (90% identity)	Epitope ID	Sub-sequence	Molecule Source Name	MHC Allele Name	Host Organism Name	Pubmed ID
D2Y4V7	283580621	Q27003	117913	HPGSVNEFDF	dense granule antigen protein 6	H-2-Ld	Mus musculus BALB/c	18587399
D2Y4V7	283580621	Q27003	117913	HPGSVNEFDF	dense granule antigen protein 6	H-2-Ld	Mus musculus B10.D2	18587399
D2Y4V7	283580621	Q27003	139947	VVFVVFMGV	dense granule antigen protein 6	HLA-A*02:01	Homo sapiens	21095258
D2Y4V7	283580621	Q27003	139747	FMGVLVNSL	dense granule antigen protein 6	HLA-A*02:01	Homo sapiens	21095258
D2Y4V7	283580621	Q27003	139747	FMGVLVNSL	dense granule antigen protein 6	HLA-A*02:01	Mus musculus HLA-A*0201/Kb Tg	21095258
D2Y4V7	283580621	Q27003	140651	AMLTAFFLR	dense granule antigen protein 6	HLA-A*11:01	Mus musculus HLA-A*1101/Kb Tg	21129215
D2Y4V7	283580621	Q27003	140651	AMLTAFFLR	dense granule antigen protein 6	HLA-A*11:01	Homo sapiens	21129215
D2Y4V7	283580621	Q27003	117913	HPGSVNEFDF	dense granule antigen protein 6	H-2-Ld	Mus musculus BALB/c	20347630
D2Y4V7	283580621	Q27003	117913	HPGSVNEFDF	dense granule antigen protein 6	H-2-Ld	Mus musculus BALB/c	20347630
D2Y4V7	283580621	Q27003	117913	HPGSVNEFDF	dense granule antigen protein 6	H-2-Ld	Mus musculus C57BL/10 X DBA/2	23818852
D2Y4V7	283580621	Q27003	117913	HPGSVNEFDF	dense granule antigen protein 6	H-2-Ld	Mus musculus C57BL/10 X DBA/2	23818852
D2Y4V7	283580621	Q27003	117913	HPGSVNEFDF	dense granule antigen protein 6	H-2-Ld	Mus musculus BALB/c	23818852
P13403	129322		10421	DTMKSMQRDED	Dense granule protein 1 precursor	allele undetermined	Mus musculus BALB/c	18555564

UniProt ID	NCBI GI	Homolog ID (90% identity)	Epitope ID	Sub-sequence	Molecule Source Name	MHC Allele Name	Host Organism Name	Pubmed ID
P13664	129348		1161	AESKSVII	Major surface antigen p30 precursor	H-2-Kk	Mus musculus C3H	10569750
P13664	129348		65781	TPTENHFTL	Major surface antigen p30 precursor	H-2-Ld	Mus musculus BALB/c	16610929
P13664	129348		63089	TCPDKKSTA	Major surface antigen p30 precursor	allele undetermined	Mus musculus BALB/c	18555564
P13664	129348		27248	ILPKLTENPWQ	Major surface antigen p30 precursor	allele undetermined	Mus musculus BALB/c	18555564
Q27002	2498423		58128	SGLTGVKDSSS	Dense granule protein 4 precursor	allele undetermined	Mus musculus BALB/c	18555564
Q27002	2498423		104255	SPMNGGYYM	Dense granule protein 4 precursor	H-2-Ld	Mus musculus BALB/c	18922097
Q27002	2498423		104255	SPMNGGYYM	Dense granule protein 4 precursor	H-2-Ld	Mus musculus BALB/c	18922097
Q27002	2498423		104255	SPMNGGYYM	Dense granule protein 4 precursor	H-2-Ld	Mus musculus BALB/c	18922097
Q27002	2498423		104255	SPMNGGYYM	Dense granule protein 4 precursor	H-2-Ld	Mus musculus C57BL/6 X DBA/2	23818852
Q27298	37778533	P13664	60031	SPEKHHCTV	SAG1 protein	HLA-A2	Homo sapiens	9240420
Q27298	37778533	P13664	60031	SPEKHHCTV	SAG1 protein	HLA-A2	Homo sapiens	9240420
Q27298	37778533	P13664	40286	LVCGKDGVK	SAG1 protein	HLA-A2	Homo sapiens	9240420
Q27298	37778533	P13664	65118	TLVCGKDGV	SAG1 protein	HLA-A2	Homo sapiens	9240420
Q27298	37778533	P13664	140697	KSFKDILPK	SAG1 protein	HLA-A*11:01	Mus musculus HLA-A*1101/Kb Tg	21129215

UniProt ID	NCBI GI	Homolog ID (90% identity)	Epitope ID	Sub-sequence	Molecule Source Name	MHC Allele Name	Host Organism Name	Pubmed ID
Q27298	37778533	P13664	140697	KSFKDILPK	SAG1 protein	HLA-A*11:01	Homo sapiens	21129215
S8EZ84	237845283	Q27003	139947	VVFVVFMGV	granule antigen protein GRA6	HLA-A*02:01	Homo sapiens	20347630
S8EZ84	237845283	Q27003	139747	FMGVLVNSL	granule antigen protein GRA6	HLA-A*02:02	Homo sapiens	20347630
S8EZ84	237845283	Q27003	140651	AMLTAFFLR	granule antigen protein GRA6	HLA-A*33:01	Homo sapiens	20347630
S8EZ84	237845283	Q27003	139947	VVFVVFMGV	granule antigen protein GRA6	HLA-A*02:03	Homo sapiens	20347630
S8EZ84	237845283	Q27003	139947	VVFVVFMGV	granule antigen protein GRA6	HLA-A*02:06	Homo sapiens	20347630
S8EZ84	237845283	Q27003	139947	VVFVVFMGV	granule antigen protein GRA6	HLA-A*68:02	Homo sapiens	20347630
S8EZ84	237845283	Q27003	139747	FMGVLVNSL	granule antigen protein GRA6	HLA-A*02:01	Homo sapiens	20347630
S8EZ84	237845283	Q27003	139747	FMGVLVNSL	granule antigen protein GRA6	HLA-A*02:03	Homo sapiens	20347630
S8EZ84	237845283	Q27003	140651	AMLTAFFLR	granule antigen protein GRA6	HLA-A*03:01	Homo sapiens	20347630
S8EZ84	237845283	Q27003	140651	AMLTAFFLR	granule antigen protein GRA6	HLA-A*11:01	Homo sapiens	20347630
S8EZ84	237845283	Q27003	140651	AMLTAFFLR	granule antigen protein GRA6	HLA-A*31:01	Homo sapiens	20347630
S8EZ84	237845283	Q27003	140651	AMLTAFFLR	granule antigen protein GRA6	HLA-A*68:01	Homo sapiens	20347630

UniProt ID	NCBI GI	Homolog ID (90% identity)	Epitope ID	Sub-sequence	Molecule Source Name	MHC Allele Name	Host Organism Name	Pubmed ID
S8F3P1	237838467	O00834	139938	VLLPVLFGV	microneme protein MIC1	HLA-A*02:01	Homo sapiens	21095258
S8F3P1	237838467	O00834	139938	VLLPVLFGV	microneme protein MIC1	HLA-A*02:02	Homo sapiens	21095258
S8F3P1	237838467	O00834	139938	VLLPVLFGV	microneme protein MIC1	HLA-A*02:03	Homo sapiens	21095258
S8F3P1	237838467	O00834	139938	VLLPVLFGV	microneme protein MIC1	HLA-A*02:06	Homo sapiens	21095258
S8F3P1	237838467	O00834	139938	VLLPVLFGV	microneme protein MIC1	HLA-A*68:02	Homo sapiens	21095258
S8F3P1	237838467	O00834	139938	VLLPVLFGV	microneme protein MIC1	HLA-A*02:01	Mus musculus HLA-A*0201/Kb Tg	21095258
S8F944	237836631	O00933	140739	RSFKDLLKK	dense granule protein 7	HLA-A*30:01	Homo sapiens	20347630
S8F944	237836631	O00933	147936	LPQFATAAT	dense granule protein 7	HLA-B*07:02	Homo sapiens	20347630
S8F944	237836631	O00933	140739	RSFKDLLKK	dense granule protein 7	HLA-A*03:01	Homo sapiens	20347630
S8F944	237836631	O00933	140739	RSFKDLLKK	dense granule protein 7	HLA-A*11:01	Homo sapiens	20347630
S8F944	237836631	O00933	140739	RSFKDLLKK	dense granule protein 7	HLA-A*31:01	Homo sapiens	20347630
S8F944	237836631	O00933	147936	LPQFATAAT	dense granule protein 7	HLA-B*42:01	Homo sapiens	20347630
S8F944	237836631	O00933	147936	LPQFATAAT	dense granule protein 7	HLA-B*54:01	Homo sapiens	20347630

4 REFERENCES

1. Kur J, Holec-Gasior L, Hiszczynska-Sawicka E (2009) Current status of toxoplasmosis vaccine development. Expert Review of Vaccines 8: 791-808.
2. Goodswen SJ, Kennedy PJ, Ellis JT (2014) Discovering a vaccine against neosporosis using computers: is it feasible? Trends in Parasitology 30: 401-411.
3. Chen Z, Harb OS, Roos DS (2008) *In Silico* Identification of Specialized Secretory-Organelle Proteins in Apicomplexan Parasites and *In Vivo* Validation in *Toxoplasma gondii*. PLoS ONE 3: e3611.
4. Louis M. Weiss KK (2014) Toxoplasma Gondii: The Model Apicomplexan - Perspectives and Methods: Academic Press.
5. Che F-Y, Madrid-Aliste C, Burd B, Zhang H, Nieves E, et al. (2010) Comprehensive proteomic analysis of membrane proteins in toxoplasma gondii. Molecular & Cellular Proteomics 10: M110 000745.
6. Jongert E, Melkebeek V, De Craeye S, Dewit J, Verhelst D, et al. (2008) An enhanced GRA1-GRA7 cocktail DNA vaccine primes anti-Toxoplasma immune responses in pigs. Vaccine 26: 1025-1031.
7. Wu X-N, Lin J, Lin X, Chen J, Chen Z-L, et al. (2012) Multicomponent DNA vaccine-encoding Toxoplasma gondii GRA1 and SAG1 primes: anti-Toxoplasma immune response in mice. Parasitology Research 111: 2001-2009.
8. Xue M, He S, Cui Y, Yao Y, Wang H (2008) Evaluation of the immune response elicited by multi-antigenic DNA vaccine expressing SAG1, ROP2 and GRA2 against Toxoplasma gondii. Parasitology International 57: 424-429.
9. Henriquez FL, Nickdel MB, McLeod R, Lyons RE, Lyons K, et al. (2005) Toxoplasma gondii dense granule protein 3 (GRA3) is a type I transmembrane protein that possesses a cytoplasmic dilysine (KKXX) endoplasmic reticulum (ER) retrieval motif. Parasitology 131: 169-179.
10. Kim JY, Ahn H-J, Ryu KJ, Nam H-W (2008) Interaction between Parasitophorous Vacuolar Membrane-associated GRA3 and Calcium Modulating Ligand of Host Cell Endoplasmic Reticulum in the Parasitism of Toxoplasma gondii. Korean Journal of Parasitology 46: 209-216.
11. Dziadek B, Gatkowska J, Grzybowski M, Dziadek J, Dzitko K, et al. (2012) Toxoplasma gondii: The vaccine potential of three trivalent antigen-cocktails composed of recombinant ROP2, ROP4, GRA4 and SAG1 proteins against chronic toxoplasmosis in BALB/c mice. Experimental Parasitology 131: 133-138.
12. Igarashi M, Kano F, Tamekuni K, Machado RZ, Navarro IT, et al. (2008) Toxoplasma gondii: Evaluation of an intranasal vaccine using recombinant proteins against brain cyst formation in BALB/c mice. Experimental Parasitology 118: 386-392.
13. Hiszczynska-Sawicka E, Oledzka G, Holec-Gasior L, Li H, Xu JB, et al. (2011) Evaluation of immune responses in sheep induced by DNA immunization with genes encoding GRA1, GRA4, GRA6 and GRA7 antigens of Toxoplasma gondii. Veterinary Parasitology 177: 281-289.
14. Sun X-M, Zou J, Elashram SAA, Yan W-C, Liu X-Y, et al. (2011) DNA vaccination with a gene encoding Toxoplasma gondii GRA6 induces partial protection against toxoplasmosis in BALB/c mice. Parasites & Vectors 4.
15. Feliu V, Vasseur V, Grover HS, Chu HH, Brown MJ, et al. (2013) Location of the CD8 T Cell Epitope within the Antigenic Precursor Determines Immunogenicity and Protection against the Toxoplasma gondii Parasite. Plos Pathogens 9.
16. El Bissati K, Zhou Y, Dasgupta D, Cobb D, Dubey JP, et al. (2014) Effectiveness of a novel immunogenic nanoparticle platform for Toxoplasma peptide vaccine in HLA transgenic mice. Vaccine 32: 3243-3248.
17. Liu Q, Wang F, Wang G, Zhao Q, Min J, et al. (2014) Toxoplasma gondii Immune response and protective efficacy induced by ROP16/GRA7 multicomponent DNA vaccine with a genetic adjuvant B7-2. Human Vaccines & Immunotherapeutics 10: 184-191.
18. Lu B, Wu ST, Shi Y, Zhang RL, Zou LJ, et al. (2006) Toxoplasma gondii: Expression pattern and detection of infection using full-length recombinant P35 antigen. Experimental Parasitology 113: 83-90.
19. Hiszczynska-Sawicka E, Li H, Xu JB, Holec-Gasior L, Kur J, et al. (2011) Modulation of immune response to Toxoplasma gondii in sheep by immunization with a DNA vaccine encoding ROP1 antigen as a fusion protein with ovine CD154. Veterinary Parasitology 183: 72-78.
20. Ossorio PN, Schwartzman JD, Boothroyd JC (1992) A Toxoplasma-gondii rhoptry protein associated with host-cell penetration has unusual charge asymmetry. Molecular and Biochemical Parasitology 50: 1-16.
21. Debache K, Alaeddine F, Guionaud C, Monney T, Mueller J, et al. (2009) Vaccination with recombinant NcROP2 combined with recombinant NcMIC1 and NcMIC3 reduces cerebral infection and vertical transmission in mice experimentally infected with Neospora caninum tachyzoites. International Journal for Parasitology 39: 1373-1384.

22. Li W-S, Chen Q-X, Ye J-X, Xie Z-X, Chen J, et al. (2011) Comparative evaluation of immunization with recombinant protein and plasmid DNA vaccines of fusion antigen ROP2 and SAG1 from Toxoplasma gondii in mice: cellular and humoral immune responses. Parasitology Research 109: 637-644.
23. Rocchi MS, Bartley PM, Inglis NF, Collantes-Fernandez E, Entrican G, et al. (2011) Selection of Neospora caninum antigens stimulating bovine CD4(+ve) T cell responses through immuno-potency screening and proteomic approaches. Veterinary Research 42: 1-91.
24. Carey KL, Jongco AM, Kam K, Ward GE (2004) The Toxoplasma gondii rhoptry protein ROP4 is secreted into the parasitophorous vacuole and becomes phosphorylated in infected cells. Eukaryotic Cell 3: 1320-1330.
25. Zheng B, Lu S, Tong Q, Kong Q, Lou D (2013) The virulence-related rhoptry protein 5 (ROP5) of Toxoplasma Gondii is a novel vaccine candidate against toxoplasmosis in mice. Vaccine 31: 4578-4584.
26. Zhao H-G, Fan Z-G, Huang F-Y, Huang Y-H, Zhou S-L, et al. (2013) Immune response in mice induced by complex gene vaccine pcSAG1-ROP5 of Toxoplasma gondii. Zhongguo ji sheng chong xue yu ji sheng chong bing za zhi = Chinese journal of parasitology & parasitic diseases 31: 1-5.
27. Parthasarathy S, Fong MY, Ramaswamy K, Lau YL (2013) Protective Immune Response in BALB/c Mice Induced by DNA Vaccine of the ROP8 gene of Toxoplasma gondii. American Journal of Tropical Medicine and Hygiene 88: 883-887.
28. Chen J, Zhou D-H, Li Z-Y, Petersen E, Huang S-Y, et al. (2014) Toxoplasma gondii: Protective immunity induced by rhoptry protein 9 (TgROP9) against acute toxoplasmosis. Experimental Parasitology 139: 42-48.
29. Wang P-Y, Yuan Z-G, Petersen E, Li J, Zhang X-X, et al. (2012) Protective Efficacy of a Toxoplasma gondii Rhoptry Protein 13 Plasmid DNA Vaccine in Mice. Clinical and Vaccine Immunology 19: 1916-1920.
30. Yuan Z-G, Zhang X-X, He X-H, Petersen E, Zhou D-H, et al. (2011) Protective Immunity Induced by Toxoplasma gondii Rhoptry Protein 16 against Toxoplasmosis in Mice. Clinical and Vaccine Immunology 18: 119-124.
31. Zhao Y, Yap GS (2014) Toxoplasma's Arms Race with the Host Interferon Response: A Menage a Trois of ROPs. Cell Host & Microbe 15: 517-518.
32. Cheng L, Chen Y, Chen L, Shen Y, Shen J, et al. (2012) Interactions between the ROP18 kinase and host cell proteins that aid in the parasitism of Toxoplasma gondii. Acta Tropica 122: 255-260.
33. El Hajj H, Lebrun M, Arold ST, Vial H, Labesse G, et al. (2007) ROP18 is a rhoptry kinase controlling the intracellular proliferation of Toxoplasma gondii. Plos Pathogens 3: 200-211.
34. Niedelman W, Gold DA, Rosowski EE, Sprokholt JK, Lim D, et al. (2012) The rhoptry proteins ROP18 and ROP5 mediate Toxoplasma gondii evasion of the murine, but not the human, interferon-gamma response. Plos Pathogens 8: e1002784-e1002784.
35. Besteiro S, Michelin A, Poncet J, Dubremetz J-F, Lebrun M (2009) Export of a Toxoplasma gondii Rhoptry Neck Protein Complex at the Host Cell Membrane to Form the Moving Junction during Invasion. Plos Pathogens 5.
36. Bradley PJ, Ward C, Cheng SJ, Alexander DL, Coller S, et al. (2005) Proteomic analysis of rhoptry organelles reveals many novel constituents for host-parasite interactions in Toxoplasma gondii. Journal of Biological Chemistry 280: 34245-34258.
37. Moreno-Perez DA, Montenegro M, Patarroyo ME, Patarroyo MA (2011) Identification, characterization and antigenicity of the Plasmodium vivax rhoptry neck protein 1 (PvRON1). Malaria Journal 10.
38. Tonkin ML, Roques M, Lamarque MH, Pugniere M, Douguet D, et al. (2011) Host Cell Invasion by Apicomplexan Parasites: Insights from the Co-Structure of AMA1 with a RON2 Peptide. Science 333: 463-467.
39. Poukchanski A, Fritz HM, Tonkin ML, Treeck M, Boulanger MJ, et al. (2013) Toxoplasma gondii Sporozoites Invade Host Cells Using Two Novel Paralogues of RON2 and AMA1. Plos One 8.
40. Ito D, Han E-T, Takeo S, Thongkukiatkul A, Otsuki H, et al. (2011) Plasmodial ortholog of Toxoplasma gondii rhoptry neck protein 3 is localized to the rhoptry body. Parasitology International 60: 132-138.
41. Takemae H, Sugi T, Kobayashi K, Gong H, Ishiwa A, et al. (2013) Characterization of the interaction between Toxoplasma gondii rhoptry neck protein 4 and host cellular beta-tubulin. Scientific Reports 3.
42. Curtidor H, Patino LC, Arevalo-Pinzon G, Patarroyo ME, Patarroyo MA (2011) Identification of the Plasmodium falciparum rhoptry neck protein 5 (PfRON5). Gene 474: 22-28.
43. Curtidor H, Patino LC, Arevalo-Pinzon G, Vanegas M, Patarroyo ME, et al. (2014) Plasmodium falciparum rhoptry neck protein 5 peptides bind to human red blood cells and inhibit parasite invasion. Peptides 53: 210-217.
44. Beck JR, Fung C, Straub KW, Coppens I, Vashisht AA, et al. (2013) A Toxoplasma Palmitoyl Acyl Transferase and the Palmitoylated Armadillo Repeat Protein TgARO Govern Apical Rhoptry Tethering and Reveal a Critical Role for the Rhoptries in Host Cell Invasion but Not Egress. Plos Pathogens 9.
45. Ortega-Barria E, Boothroyd JC (1999) A Toxoplasma lectin-like activity specific for sulfated polysaccharides is involved in host cell infection. Journal of Biological Chemistry 274: 1267-1276.

46. Garcia-Reguet N, Lebrun M, Fourmaux MN, Mercereau-Puijalon O, Mann T, et al. (2000) The microneme protein MIC3 of Toxoplasma gondii is a secretory adhesin that binds to both the surface of the host cells and the surface of the parasite. Cellular Microbiology 2: 353-364.
47. Ismael AB, Sekkai D, Collin C, Bout D, Mevelec MN (2003) The MIC3 gene of Toxoplasma gondii is a novel potent vaccine candidate against toxoplasmosis. Infection and Immunity 71: 6222-6228.
48. Lourenco EV, Panunto-Castelo A, Molfetta JB, Avanci NC, Goldman MHS, et al. (2006) Immunization with MIC1 and MIC4 induces protective immunity against toxoplasma gondii. Glycobiology 16: 1112-1112.
49. Peng GH, Yuan ZG, Zhou DH, He XH, Yan C, et al. (2010) Sequence Variation in Toxoplasma gondii MIC4 Gene and Protective Effect of an MIC4 DNA Vaccine in a Murine Model Against Toxoplasmosis. Journal of Animal and Veterinary Advances 9: 1463-1468.
50. Wang H, He S, Yao Y, Cong H, Zhao H, et al. (2009) Toxoplasma gondii: Protective effect of an intranasal SAG1 and MIC4 DNA vaccine in mice. Experimental Parasitology 122: 226-232.
51. Peng G-H, Yuan Z-G, Zhou D-H, He X-H, Liu M-M, et al. (2009) Toxoplasma gondii microneme protein 6 (MIC6) is a potential vaccine candidate against toxoplasmosis in mice. Vaccine 27: 6570-6574.
52. Yan H-K, Yuan Z-G, Song H-Q, Petersen E, Zhou Y, et al. (2012) Vaccination with a DNA Vaccine Coding for Perforin-Like Protein 1 and MIC6 Induces Significant Protective Immunity against Toxoplasma gondii. Clinical and Vaccine Immunology 19: 684-689.
53. Liu MM, Yuan ZG, Peng GH, Zhou DH, He XH, et al. (2010) Toxoplasma gondii microneme protein 8 (MIC8) is a potential vaccine candidate against toxoplasmosis. Parasitology Research 106: 1079-1084.
54. Yao Y, He S-y, Wang H-x, Zhou H-y, Zhao H, et al. (2010) Protective immunity induced in mice by multiantigenic DNA vaccine with genes encoding SAG1 and MIC8 of Toxoplasma gondii. Zhongguo ji sheng chong xue yu ji sheng chong bing za zhi = Chinese journal of parasitology & parasitic diseases 28: 81-88.
55. Zhao H-G, Huang F-Y, Guo J-L, Tan G-H (2013) Evaluation on the immune response induced by DNA vaccine encoding MIC8 co-immunized with IL-12 genetic adjuvant against Toxoplasma gondii infection. Zhongguo ji sheng chong xue yu ji sheng chong bing za zhi = Chinese journal of parasitology & parasitic diseases 31: 284-289.
56. Li Z-Y, Chen J, Petersen E, Zhou D-H, Huang S-Y, et al. (2014) Synergy of mIL-21 and mIL-15 in enhancing DNA vaccine efficacy against acute and chronic Toxoplasma gondii infection in mice. Vaccine 32: 3058-3065.
57. Harper JM, Zhou XW, Pszenny V, Kafsack BFC, Carruthers VB (2004) The novel coccidian micronemal protein MIC11 undergoes proteolytic maturation by sequential cleavage to remove an internal propeptide. International Journal for Parasitology 34: 1047-1058.
58. Ren D, Zhou D-H, Xu M-J, Zhou Y, Yang J-F, et al. (2012) Sequence variation in Toxoplasma gondii MIC13 gene among isolates from different hosts and geographical locations. African Journal of Microbiology Research 6: 3265-3269.
59. Yuan Z-G, Ren D, Zhou D-H, Zhang X-X, Petersen E, et al. (2013) Evaluation of protective effect of pVAX-TgMIC13 plasmid against acute and chronic Toxoplasma gondii infection in a murine model. Vaccine 31: 3135-3139.
60. Yan H-K, Yuan Z-G, Petersen E, Zhang X-X, Zhou D-H, et al. (2011) Toxoplasma gondii: Protective immunity against experimental toxoplasmosis induced by a DNA vaccine encoding the perforin-like protein 1. Experimental Parasitology 128: 38-43.
61. Tan F, Hu X, Luo F-J, Pan C-W, Chen X-G (2011) Induction of protective Th1 immune responses in mice by vaccination with recombinant Toxoplasma gondii nucleoside triphosphate hydrolase-II. Vaccine 29: 2742-2748.
62. Bulow R, Boothroyd JC (1991) Protection of mice from fatal *Toxoplasma-gondii* infection by immunization with p-30 antigen in liposomes. Journal of Immunology 147: 3496-3500.
63. Fux B, Rodrigues CV, Portela RW, Silva NM, Su CL, et al. (2003) Role of cytokines and major histocompatibility complex restriction in mouse resistance to infection with a natural recombinant strain (type I-III) of Toxoplasma gondii. Infection and Immunity 71: 6392-6401.
64. Kasper LH, Khan IA, Ely KH, Buelow R, Boothroyd JC (1992) Antigen-specific (p-30) mouse CD8+ T-cells are cytotoxic against Toxoplasma-gondii-infected peritoneal-macrophages. Journal of Immunology 148: 1493-1498.
65. Khosroshahi KH, Ghaffarifar F, D'Souza S, Sharifi Z, Dalimi A (2011) Evaluation of the immune response induced by DNA vaccine cocktail expressing complete SAG1 and ROP2 genes against toxoplasmosis. Vaccine 29: 778-783.
66. Haldorson GJ, Mathison BA, Wenberg K, Conrad PA, Dubey JP, et al. (2005) Immunization with native surface protein NcSRS2 induces a Th2 immune response and reduces congenital Neospora caninum transmission in mice. International Journal for Parasitology 35: 1407-1415.

67. Howe DK, Crawford AC, Lindsay D, Sibley LD (1998) The p29 and p35 immunodominant antigens of Neospora caninum tachyzoites are homologous to the family of surface antigens of Toxoplasma gondii. Infection and Immunity 66: 5322-5328.
68. Wasmuth JD, Pszenny V, Haile S, Jansen EM, Gast AT, et al. (2012) Integrated Bioinformatic and Targeted Deletion Analyses of the SRS Gene Superfamily Identify SRS29C as a Negative Regulator of Toxoplasma Virulence. Mbio 3.
69. Lau YL, Fong MY (2008) Toxoplasma gondii: Serological characterization and immunogenicity of recombinant surface antigen 2 (SAG2) expressed in the yeast Pichia pastoris. Experimental Parasitology 119: 373-378.
70. Li J-h, Wu S-t, Weng Y-b, Gan Y, Liu H-b, et al. (2007) Study on immuno-effect with GRA4 or SAG2 gene recombinant BCG vaccine of Toxoplasma gondii. Zhongguo ji sheng chong xue yu ji sheng chong bing za zhi = Chinese journal of parasitology & parasitic diseases 25: 217-221.
71. Machado AV, Caetano BC, Barbosa RP, Salgado APC, Rabelo RH, et al. (2010) Prime and boost immunization with influenza and adenovirus encoding the Toxoplasma gondii surface antigen 2 (SAG2) induces strong protective immunity. Vaccine 28: 3247-3256.
72. Lekutis C, Ferguson DJP, Grigg ME, Camps M, Boothroyd JC (2001) Surface antigens of Toxoplasma gondii: variations on a theme. International Journal for Parasitology 31: 1285-1292.
73. Lekutis C, Ferguson DJP, Boothroyd JC (2000) Toxoplasma gondii: Identification of a developmentally regulated family of genes related to SAG2. Experimental Parasitology 96: 89-96.
74. Zhang M, Zhao L, Song J, Li Y, Zhao Q, et al. (2013) DNA vaccine encoding the Toxoplasma gondii bradyzoite-specific surface antigens SAG2CDX protect BALB/c mice against type II parasite infection. Vaccine 31: 4536-4540.
75. Cong H, Yuan Q, Zhao Q, Zhao L, Yin H, et al. (2014) Comparative efficacy of a multi-epitope DNA vaccine via intranasal, peroral, and intramuscular delivery against lethal Toxoplasma gondii infection in mice. Parasites & Vectors 7.
76. Cong H, Zhang M, Xin Q, Wang Z, Li Y, et al. (2013) Compound DNA vaccine encoding SAG1/SAG3 with A(2)/B subunit of cholera toxin as a genetic adjuvant protects BALB/c mice against Toxoplasma gondii. Parasites & Vectors 6.
77. Spano F, Ricci I, Di Cristina M, Possenti A, Tinti M, et al. (2002) The SAG5 locus of Toxoplasma gondii encodes three novel proteins belonging to the SAG1 family of surface antigens. International Journal for Parasitology 32: 121-131.
78. Tinti M, Possenti A, Cherchi S, Barca S, Spano F (2003) Analysis of the SAG5 locus reveals a distinct genomic organisation in virulent and avirulent strains of Toxoplasma gondii. International Journal for Parasitology 33: 1605-1616.
79. McAllister MM, Parmley SF, Weiss LM, Welch YJ, McGuire AM (1996) An immunohistochemical method for detecting bradyzoite antigen (BAGS) in Toxoplasma gondii-infected tissues cross-reacts with a Neospora caninum bradyzoite antigen. Journal of Parasitology 82: 354-355.
80. Wang Q, Wu K, Chen X-g, Hao L, Cheng L (2007) Cloning and expression of a bradyzoite-specific gene of Toxoplasma gondii and immunoreactive analysis on the recombinant antigen. Zhongguo ji sheng chong xue yu ji sheng chong bing za zhi = Chinese journal of parasitology & parasitic diseases 25: 295-299.
81. Crawford J, Grujic O, Bruic E, Czjzek M, Grigg ME, et al. (2009) Structural Characterization of the Bradyzoite Surface Antigen (BSR4) from Toxoplasma gondii, a Unique Addition to the Surface Antigen Glycoprotein 1-related Superfamily. Journal of Biological Chemistry 284: 9192-9198.
82. Yin L-T, Wang F, Meng X-L, Wang H-L, Liu H-L, et al. (2012) Cloning, expression and antigenicity analysis of phosphoglycerate mutase 2 gene of Toxoplasma gondii. Zhongguo ji sheng chong xue yu ji sheng chong bing za zhi = Chinese journal of parasitology & parasitic diseases 30: 86-89.
83. Saadatnia G, Mohamed Z, Ghaffarifar F, Osman E, Moghadam ZK, et al. (2012) Toxoplasma gondii excretory secretory antigenic proteins of diagnostic potential. Apmis 120: 47-55.
84. Hemphill A, Gottstein B, Kaufmann H (1996) Adhesion and invasion of bovine endothelial cells by Neospora caninum. Parasitology 112: 183-197.
85. Howe DK, Sibley LD (1999) Comparison of the major antigens of Neospora caninum and Toxoplasma gondii. International Journal for Parasitology 29: 1489-1496.
86. Carruthers V, Boothroyd JC (2007) Pulling together: an integrated model of Toxoplasma cell invasion. Current Opinion in Microbiology 10: 83-89.
87. Mercier C, Adjogble KDZ, Daubener W, Delauw MFC (2005) Dense granules: Are they key organelles to help understand the parasitophorous vacuole of all apicomplexa parasites? International Journal for Parasitology 35: 829-849.
88. Braun L, Travier L, Kieffer S, Musset K, Garin J, et al. (2008) Purification of Toxoplasma dense granule proteins reveals that they are in complexes throughout the secretory pathway. Molecular and Biochemical Parasitology 157: 13-21.

89. Lang C, Gross U, Lueder CGK (2007) Subversion of innate and adaptive immune responses by Toxoplasma Gondii. Parasitology Research 100: 191-203.
90. Sonda S, Fuchs N, Connolly B, Fernandez P, Gottstein B, et al. (1998) The major 36 kDa Neospora caninum tachyzoite surface protein is closely related to the major Toxoplasma gondii surface antigen. Molecular and Biochemical Parasitology 97: 97-108.
91. Hemphill A, Fuchs N, Sonda S, Gottstein B, Hentrich B (1997) Identification and partial characterization of a 36 kDa surface protein on Neospora caninum tachyzoites. Parasitology 115: 371-380.
92. Nielsen HV, Lauemoller SL, Christiansen L, Buus S, Fomsgaard A, et al. (1999) Complete protection against lethal Toxoplasma gondii infection in mice immunized with a plasmid encoding the SAG1 gene. Infection and Immunity 67: 6358-6363.
93. Liu K-Y, Zhang D-B, Wei Q-K, Li J, Li G-P, et al. (2006) Biological role of surface Toxoplasma gondii antigen in development of vaccine. World Journal of Gastroenterology 12: 2363-2368.
94. Jung C, Lee CYF, Grigg ME (2004) The SRS superfamily of Toxoplasma surface proteins. International Journal for Parasitology 34: 285-296.
95. Hemphill A (1996) Subcellular localization and functional characterization of Nc-p43, a major Neospora caninum tachyzoite surface protein. Infection and Immunity 64: 4279-4287.
96. Hemphill A, Felleisen R, Connolly B, Gottstein B, Hentrich B, et al. (1997) Characterization of a cDNA-clone encoding Nc-p43, a major Neospora caninum tachyzoite surface protein. Parasitology 115: 581-590.
97. Haldorson GJ, Stanton JB, Mathison BA, Suarez CE, Baszler TV (2006) Neospora caninum: Antibodies directed against tachyzoite surface protein NcSRS2 inhibit parasite attachment and invasion of placental trophoblasts in vitro. Experimental Parasitology 112: 172-178.
98. Ellis JT, Ryce C, Atkinson R, Balu S, Jones P, et al. (2000) Isolation, characterization and expression of a GRA2 homologue from Neospora caninum. Parasitology 120: 383-390.
99. Hiszczynska-Sawicka E, Li H, Xu JB, Oledzka G, Kur J, et al. (2010) Comparison of immune response in sheep immunized with DNA vaccine encoding Toxoplasma gondii GRA7 antigen in different adjuvant formulations. Experimental Parasitology 124: 365-372.
100. Dubremetz JF, Garcia-Reguet N, Conseil V, Fourmaux MN (1998) Apical organelles and host-cell invasion by Apicomplexa. International Journal for Parasitology 28: 1007-1013.
101. Chen Z, Harb OS, Roos DS (2008) In Silico Identification of Specialized Secretory-Organelle Proteins in Apicomplexan Parasites and In Vivo Validation in Toxoplasma gondii. PLoS ONE 3.
102. Debache K, Guionaud C, Alaeddine F, Mevissen M, Hemphill A (2008) Vaccination of mice with recombinant NcROP2 antigen reduces mortality and cerebral infection in mice infected with Neospora caninum tachyzoites. International Journal for Parasitology 38: 1455-1463.
